# SorghumFDB: sorghum functional genomics database with multidimensional network analysis

**DOI:** 10.1093/database/baw099

**Published:** 2016-06-27

**Authors:** Tian Tian, Qi You, Liwei Zhang, Xin Yi, Hengyu Yan, Wenying Xu, Zhen Su

**Affiliations:** State Key Laboratory of Plant Physiology and Biochemistry, College of Biological Sciences, China Agricultural University, Beijing 100193, China

## Abstract

Sorghum (*Sorghum bicolor* [L.] Moench) has excellent agronomic traits and biological properties, such as heat and drought-tolerance. It is a C_4_ grass and potential bioenergy-producing plant, which makes it an important crop worldwide. With the sorghum genome sequence released, it is essential to establish a sorghum functional genomics data mining platform. We collected genomic data and some functional annotations to construct a sorghum functional genomics database (SorghumFDB). SorghumFDB integrated knowledge of sorghum gene family classifications (transcription regulators/factors, carbohydrate-active enzymes, protein kinases, ubiquitins, cytochrome P450, monolignol biosynthesis related enzymes, R-genes and organelle-genes), detailed gene annotations, miRNA and target gene information, orthologous pairs in the model plants *Arabidopsis*, rice and maize, gene loci conversions and a genome browser. We further constructed a dynamic network of multidimensional biological relationships, comprised of the co-expression data, protein–protein interactions and miRNA-target pairs. We took effective measures to combine the network, gene set enrichment and motif analyses to determine the key regulators that participate in related metabolic pathways, such as the lignin pathway, which is a major biological process in bioenergy-producing plants.

**Database URL:** http://structuralbiology.cau.edu.cn/sorghum/index.html.

## Introduction

Sorghum originated from Africa and is the 5th major cereal in terms of production and acreage throughout the world. It occupies 8 million hectares of farmland and provides food, feed, fiber and fuel. According to the Consultative Group for International Agricultural Research’s report in 2010, the USA was the world’s largest producer of sorghum (8.8 million metric tons annually), followed by India (7.0), Mexico (6.9), Nigeria (4.8) and Argentina (3.6). The excellent agronomic traits and biological properties, such as heat and drought-tolerant, of sorghum, a C_4_ grass and potential bioenergy-producing plant, make it an important crop around the world. Sorghum can be classified into several landraces with different levels of genetic diversity. Among them, three uses, grain, forage and sweet sorghum, based on work by Chromatin, Inc. (http://www.chromatininc.com/), attract a great number of sorghum experts. Grain sorghum has a high content of starch and can supply nutrition to humans and animals; forage sorghum is highly digestible and used as animal feed or as a cover crop; Sweet sorghum has a high content of sugar. These three kinds of sorghum can be used as raw materials for the industrial production of ethanol or other specialty chemicals.

In addition to maize and millet, sorghum is a common bioenergy-producing plant, as well as a C_4_ plant. Biomass is an organic carrier that transfers solar energy to biomass energy through photosynthesis (1, 2). Bioenergy-producing plants have characteristics like stress resistance, large size, quick growth and high productivity, and also higher contents of cellulose, hemicellulose, lignin, sugar, starch and grease. Sorghum is considered a source of biomass for the production of bioenergy and biofuels, as well as for use in the chemical industry (3). Nonetheless, the depth of research and yields in sorghum were more limited than in other grain crops, such as maize, rice and wheat. As an important bioenergy-producing plant, sorghum provides large amount of cellulose, which can be transformed to bioethanol through biological, physical and chemical methods (4, 5). However, because the mixture of lignin and cellulose reduces the conversion efficiency, lowering the lignin content or changing the lignin structure can enhance biomass availability (1, 6). At present, bioengineering methods have been applied to changing the activity of related enzymes, such as cinnamyl alcohol dehydrogenase (CAD), caffeic acid O-methyltransferase and ferulic acid/coniferaldehyde/coniferyl alcohol 5-hydroxylase, to alter the content or structure of lignin, making it more easily degradable (6, 7). However, the whole lignin pathway is still unclear. Discoveries and modifications of suitable enzymes cannot only accelerate biomass usage, but also enhance plant growth (2). Thus, the identification and functional analyses of related regulatory genes through the whole genome network may aid studies on sorghum.

The *Sorghum bicolor* BTx623 genome sequencing result was published in 2009 (8). Fundamental annotations of gene structures and other information on that assembly were later improved (version 1.0, 1.4 and 2.1), with more accurate parameters, comprehensive methods and a newly integrated dataset. Recent studies have made great progress in the bioenergy production, genetic variation, regulatory factors and metabolic pathways of sorghum under the sequenced genome’s background. For example, some synthesis databases have added sorghum information, including Phytozome (9), Gramene (10), National Center for Biotechnology Information (NCBI), PLAZA (11) and PlantsDB (12). MOROKOSHI (13), a sorghum transcriptome database, integrated functional annotations and used specific RNA-seq data to construct a co-expression network and further study expression profile variations.

Since the development of the microarray and next generation sequencing technology, more and more transcriptome data has become available. Prior to December 2015, the Gene Expression Omnibus (GEO, http://www.ncbi.nlm.nih.gov/geo/) in NCBI had collected 17 series, 16 platforms and 177 samples for sorghum, including GSE50464 (14), GSE54705 (15) and GSE49879 (16), and their data types covered mRNA-seq, ncRNA-seq, microarrays and others. GSE49879 contains 78 samples of microarray data, which was from six genotypes (AR2400, Atlas, Fremont, PI152611 and PI455230) and four tissues (leaf, root, shoot and stem). Thus, it was possible to construct a whole genome co-expression network. In addition, the predicted sorghum protein–protein interactions (PPIs) from the experimentally validated plant data were also useful for predicting sorghum gene function annotations.

Due to the information provided by related databases and public papers, it was necessary to build a sorghum database for research workers using a more comprehensive search criteria and analysis, similar to the popular single species functional genomic databases in agricultural, such as TIGR, MaizeGDB and SIFGD. Driven by this need, we built a comprehensive platform for the genome functional annotation of sorghum, which was named as the sorghum genomics functional database (SorghumFDB). It contains eight gene family categories, super families for transcription factors/regulators (TFs/TRs), carbohydrate-active enzymes (CAZymes), protein kinases (PKs), ubiquitins (UBs), cytochrome P450 members (CYPs), monolignol biosynthesis (MBs) related protein coding genes, R-genes and organelle-genes. In addition, detailed gene annotations, miRNA and target mRNA information, orthologous relationships with *Arabidopsis*, maize and rice, gene loci conversions and a genome browser, as well as some analysis tools, such as gene set enrichment analysis (GSEA), motif significance analysis and pattern set tools. Users can visit SorghumFDB at the URL http://structuralbiology.cau.edu.cn/sorghum/. With the accessible information and online tools mentioned, we hope that SorghumFDB will aid sorghum functional genomics analyses and become useful for sorghum and other bioenergy plant-related studies.

## Data source

The SorghumFDB was mainly built on version 2.1, which contained 33 032 genes, 39 441 transcripts and other annotation information, including the eukaryote-specific version of the Clusters of Orthologous Groups (KOG) (17) and gene ontology (GO) (18). The NCBI version of the sorghum organelle genome annotation was composed of NC_008602.1 for the chloroplast and NC_008360.1 for the mitochondrial genome. We collected miRNAs from miRBase (19) and PMRD (20). Single nucleotide polymorphisms (SNPs), expressed sequence tags (ESTs) and motifs located in the nucleic genome sequence may regulate gene expression, directly or indirectly. Sorghum SNP data were collected from SorGSD (http://sorgsd.big.ac.cn/snp/index.jsp). EST and repeats were downloaded from Phytozome v10. Combining text-mining technology with data from plantCARE (21), PLACE (22) and AthMap (23), we collected 930 motifs with publication annotations. The orthologous pairs between sorghum and its related species (rice and maize) were derived from Gramene (10). We focused mainly on TFs/TRs and seven other super families, CAZymes, PKs, UBs, CYPs, MBs, R-genes and organelle-genes. The TFs/TRs were collected from PlantTFDB (24) and PlnTFDB (25). The cytochrome P450 family members were collected from Nelson *et al*. (26). The ubiquitin families were downloaded from UUCD (27). The monolignol biosynthesis families and R-genes were collected by text-mining from Shakoor *et al*. (16) and Mace *et al*. (28), respectively. To strengthen the functional annotations, we provided GO (18), Uniprot, Panther (29) and KOG (17) annotations for most of the genes. To further analyse the relationships between genes or protein pairs, the experimentally validated plant PPI data were collected from databases, IntAct (30), Biogrid (31), iMEX (32), TAIR (33), BAR (34), CCSB (35) and published papers (36). Because of the lack of sorghum PPI data, we first collected data from maize and rice, which have close relative relationships with sorghum, but the resulting gene pairs were too few to construct a network. Then, we chose the model plant *Arabidopsis* and adapted more stringent filter criteria. Together with the orthologous pairs this data was used to build the predicted PPI network. The transcriptome data GSE50464 (14) and GSE54705 (15) were used to create the expression profile tendency chart, and GSE49879 (16) was selected to be used to construct the co-expression network.

## Construction

### Functional annotation

In the gene detail page of the SorghumFDB, there are many functional module annotations, such as KOG (17), Panther (29), domain, SNP, GO (18), Uniprot and pathway. The UniProt database has abundant information and extensive protein resources. We determined the Uniprot IDs and annotations for the genes. Additionally, the Panther (29) classification system was designed to classify proteins based on family, pathway, molecular function and biological process. We downloaded the *S. bicolor* information from Panther (29) and incorporated it into our gene detail. The KOG (17) annotation was collected from Phytozome and linked to the NCBI conserved domain database, which consists of a collection of well-annotated multiple sequence alignment models for ancient domains and full-length proteins. The protein domains were predicted using the PfamScan software from the Pfam database (37). SNP sites located either in the 2 kb upstream of the gene transcript start site or gene body region could be linked to SorGDB (http://sorgsd.big.ac.cn/snp/index.jsp), a collection of *S. bicolor* SNPs, and visualized by Gbrowse. Different databases also use specialized gene identifiers or distinct versions to define the same gene sequence. Therefore, we transformed other gene names to the uniform version 2.1 for a broader annotation and user convenience. The KEGG pathway annotation of sorghum proteins contained 131 kinds of metabolic processes, but 433 enzymes could not be mapped to version 2.1. Then, we use the BLASTP algorithm to compare the protein sequences to version 2.1, while considering whether the two sequences have the same domains predicted by Pfam (37). Finally, we identified 4399 enzymes and 131 pathways from KEGG. PlantCyc (38) provides a broad network of plant metabolic pathway databases that contain curated information from the literature and a computational analysis of the genes, enzymes, compounds, reactions and pathways involved in plant primary and secondary metabolism. We downloaded the SorghumBicolorCyc 3.0 (38) dataset and gained 3377 annotated genes and 535 pathways.

### Gene family classification

Because of the limited functional annotations, it is essential to predict potential gene functions for sorghum through comparative genomics and related algorithms. At present, we mainly perform the super family classifications for eight gene categories (39, 40), TFs/TRs, CAZymes, PKs, UBs, CYPs, MBs enzymes, R-genes and organelle-genes, which perform remarkable functions in biological processes. In addition to the eight important super families, we will add new gene families constantly as the family information is published, as revealed by new software and even for specific user requirements. Sorghum is a C_4_ model plant that can use solar energy with high-efficiency during photosynthesis. The chloroplast is part of main reaction site for C_4_ photosynthesis. Thus, integrating related carbohydrate-active enzymes and organelle-genes will aid research on the engineering of C_4_ photosynthesis and in C_3_ crops (41). In particular, the enzymes from the monolignol biosynthesis gene family have important functions in the lignin pathway. CYPs act on the production of structural components, light harvesting and hormone biosynthesis (26). They can be coupled directly to the photosynthetic energy output to obtain an environmentally friendly production of complex chemical compounds. In addition, they also participate in the biosynthesis of physiologically important compounds, such as fatty acids and steroid hormones (42). TFs/TRs, PKs and UBs participate in many signal transduction processes corresponding to biotic and abiotic stress, such as drought stress (43–45). Disease resistance is the main target of sorghum genetic improvement and contributes to high yield and quality. With the development of sorghum genome research, a large number of sorghum resistance genes have been found and located on the genetic linkage map (46).

We have three main analysis strategies: (i) comparative genomics based on the BLASTP algorithm and Inparanoid (47) software, (ii) annotation data collected directly from corresponding databases or published papers and (iii) predictions using certain software, such as iTAK (48) with the Hidden Markov Model (HMM) constructed by specific protein domain rules.

### miRNA

We have collected 223 mature miRNAs with 241 precursor sequences (-5p and -3p) in miRBase (19) Release 21. Still 173 miRNAs in PMRD (20) and only three mature miRNA (sbi-miR165, sbi-miR169l and sbi-miR157) sequences cannot be found in miRBase. We used the GMAP version 2015-11-20 (49) software map to try and identify the three precursor sequences in the sorghum genome, but only sbi-miR169l matched. The locational information on the chromosome was well annotated. We also predicted the target mRNAs of miRNA using psRNAtarget (50), a plant small RNA target prediction toolkit using a proven scoring scheme that calculates unpaired energy (UPE) online. We obtained the expression profiles of 82 miRNAs using the miRNA-seq data GSE32458 from PNRD and 47 miRNAs from the microarray data series GSE49879 to determine the expression trends.

### Network

The multidimensional network, including PPIs, co-expression relationships between genes (including miRNAs) and miRNA–target pairs (Figure 2E). The sorghum genome-wide network could help us understand the relationships between molecules.

(1) Predicted PPI network

The PPI network was based on all of the available experimentally verified PPI pairs in *Arabidopsis*, maize and rice, as well as sorghum, from IntAct (30), Biogrid (31), iMEX (32), TAIR (33), BAR (34), CCSB (35) and eTRAIN (36) owing to the deficiency in the predicted sorghum PPI network (Table 1). Although the relationship between *Arabidopsis* and sorghum is not close, the PPI pairs are too limited to construct the network only using maize and rice. Therefore, we chose relatively strict parameters, with up to 60% bootstrap support, produced by Inparanoid (47) software to determine orthologs. We used the comparative genomics method to map the PPIs of other plants to sorghum PPIs.

**Table 1.** Summary of the data sources

Category	Description	Source/method	Details
Genome	version 2.1	Phytozome v10	33032
	version 1.4	Phytozome v9	27608
	version 1	plantsDB	34567
	Organelle	NCBI	194
	miRNA	miRBase, PMRD	242
Sequence element	SNP	SorGSB	∼62 million
	Repeat masker	Phytozome v10	639245
	EST	Phytozome v10	∼404650
	Motif	plantCARE, PLACE, AthMap	930
Transcriptome	RNA-seq	NCBI/GEO	GSE50464, GSE54709, SRP008469
	Microarray	NCBI/GEO	GSE49879
Interaction	protein–protein interaction	Biogrid, iMEX, Intact TAIR, CCSB, ETRAIN	3871 genes, 11621 pairs
	co-expression interaction	PCC,MR	144,901 positive pairs
			136,596 negative pairs
	miRNA-targets	psRNAtarget	4,376 pairs
Functional annotation	KEGG pathway	KEGG	4399 genes, 131 pathways
	plantCyc pathway	plantCyc	3377 genes, 535 pathways
	KOG	Phytozome v10	3150
	Panther	Panther	2836
	Uniprot	UniProt	32796
	GO	Phytozome v10, Gramene	1177
	Domain	Pfam	3832
	Ortholog	*Arabidopsis*	111,461 pairs
		rice	21,176 pairs
		maize	19,691 pairs
Gene family	Transcription regulators/factors	plantTFDB, plnTFDB	2541genes,85 families
	Carbohydrate-active enzymes	CAZy	1166 genes, 6 classes, 99 families
	Protein kinases	PCC database, iTAK	1253genes,84 families
	Ubiquitins	UUCD	1846 genes,35 families
	Cytochrome P450	Nelson.et al.	176 genes, 81 families
	Monolignol biosynthesis enzymes	Shakoor et al.	36 genes,10 families
	R-genes	Mace et al.	231 genes,12 classes
	Organelle-genes	NCBI	194 elements, 2 families

(2) Co-expression network

Since the development of next generation sequencing technologies, large amounts of ‘omics’ data, like RNA-seq and microarray, have become available on public platforms (e.g. GEO and SRA). A large number of probes arranged on a microarray can represent the whole or part of a genome. By RNA extraction, followed by reverse transcription to cDNA, and then fluorescence labeling, we obtained the expression quantity through the fluorescent signals. While the RNA-seq technology is much easier, it can reveal unknown transcripts. The RNA-seq dataset displays the gene expression trend in different tissues and after different treatments in our gene detail webpage. We only chose the microarray data GSE49879 (Supplementary Table S1) that contains 78 samples from a combination of four different tissues (shoot, root, leaf and stem), two dissected stem tissues (pith and rind) and six diverse lines (R159, Atlas, Fremont, PI152611, AR2400 and PI455230) among three landraces (sweet, grain and forage) to construct the co-expression network, as well as genotype-specific and tissue-preferential networks. The computational process (51) was described as follows: First, if a gene matched with more than one probe set, then the highest normalized intensity among those probe sets was selected as its expression value. The screened data was maintained for the next step. Second, we filtered the data with a strict cutoff [max–min = 0.58, which is log_2_(1.5)] to prevent housekeeping genes from making background noise. Third, Pearson’s correlation coefficient (PCC) scores between genes or miRNAs were computed to evaluate their expression relationships. To confirm the correction, we used mutual rank (MR), which showed a higher prediction efficiency than PCC values by taking a geometric average of the PCC rank (52, 53). Finally, we filtered the interaction pairs and divided them into three levels, the first level contained genes having the top three PCCs; the second level contained genes with the MR ≤ 5, and the third level kept the ones with the 5 < MR ≤ 30 (Supplementary Figure S3). For the network, we set a cut-off value of 4.56. The gene is specifically expressed if the expression value is higher than 4.56 in any of the repeats or is not expressed if the expression value is lower than 4.56 in all of the repeats under one condition.

PCC calculates the linear correlation between two variables, X and Y, using the following formula:

$$PCC=\frac{\sum_{i=1}^{n}\left(x_{i}-\overset{-}{x}\right)\left(y_{i}-\overset{-}{y}\right)}{\sqrt{\sum_{i=1}^{n}{\left(x_{i}-\overset{-}{x}\right)}^{2}\cdot \sum_{i=1}^{n}{\left(y_{i}-\overset{-}{y}\right)}^{2}}}$$

Here, *X* and *Y* represent the expression quantities of two genes. The *i* indicates different samples under various conditions or times (Supplementary Figure S2).

The MR values were calculated using the following computational formula:

$$MR=\sqrt{ab}$$

For two genes, *X* and *Y*, selecting all of the pairs related to gene *X* and ranking the PCC from high to low, the position (starting from 1, with a step size of 1) of the *Y* gene is represented by *a*, while *b* represents the position of *X* in *Y*’s PCC list.

(3) miRNA-target network

The miRNA–target pairs were computed by psRNAtarget (50) through base pairing of miRNAs with their complementary mRNA targets. miRNAs play dominant roles in post-transcriptional gene regulation and have been experimentally proven to greatly affect crop plant productivity and quality (54).

(4) Functional annotation for each gene

We retrieved the single gene annotation from Phytozome, and it is displayed by clicking on the nodes in the network. A detailed gene list appears below, containing PCC, MR and PPI sources with links.

(5) Functional module prediction and annotation

After filtering false positive gene pairs based on the MR method, we had 281 497 remaining gene pairs. We used Cfinder (55), a free software based on the Clique Percolation Method, to find overlapping dense groups of nodes in the co-expression network. Using a balance between gene coverage and overlapping rates among modules, we selected the result when *k* = 5 (Supplementary Figure S4) as the threshold, which meant that a module must have at least five nodes based on the gene coverage degree, community distribution and overlap rate, and then we filtered the communities that contained more than 500 nodes. Finally, we selected 987 modules contained 3954 genes. Each network module was subjected to a GSEA analysis (*P*-value ≤ 0.05 and false discovery rate ≤ 0.05) to annotate the models.

## Tools

Motifs (cis-regulatory elements) are a series of short conserved sequences in the promoter region. They can be recognized and bound by TFs/TR and then participate together in the regulation of downstream genes. With identified motifs and published annotations, we predicted TF/TR binding regions, which could benefit studies on the relationships between TFs and their target genes. In addition to scanning for motif sequences, we offered a *Z*-score method to calculate the significance of enriched motifs (39). All of the sorghum genes’ promoters have been calculated as background, and users can submit gene sequences or gene lists, in which they can search for related motifs and carry out motif enrichment analyses.

$$Z=\frac{N_{{motif}_{i}}-{mean}_{{motif}_{i}}}{{stdev}_{{motif}_{i}}}$$

$$p-value=1-pnorm(N_{{motif}_{i}},{mean}_{{motif}_{i}},{stdev}_{{motif}_{i}})$$

where *N*_motif__*i*_ indicates the number of occurrences of motif_*i*_ in your promoters, mean_motif__*i*_ indicates the average occurrences of motif_*i*_ in these 1000 sets, stdev_motif__*i*_ indicates their standard deviation and pnorm indicates the distribution function for the normal distribution in the R language (56).

The pattern set tool also uses similar algorithms as in the motif analysis. We included the transcriptome datasets GSE50464, GSE54705, GSE49879 and SRP008469, which contain samples from different tissues, treatments, and with different genotypes as background. Users can set specific expression levels, ranked as high, even and low in each sample, indicating specific expression patterns in a data series. Then, users can obtain the screening gene list based on their settings (51).

(a) $$z=\frac{\overset{-}{x}-\overset{=}{x}}{stdev}\;$$

(b) $$z=\log_{2}\frac{{\overset{-}{x}}_{T}}{{\overset{-}{x}}_{C}}\;$$

where $\overset{-}{x}$ represents the average expression value of the repetitions for one gene, $\overset{=}{x}$ represents the average expression value of all of the samples for one dataset, *stdev* represents the standard deviation of all of the samples for one dataset; and ${\overset{-}{x}}_{T}$ represents the average expression value of the treated samples, ${\overset{-}{x}}_{C}$ represents the average expression value of the control samples. The (*a*) indicates a tissue-specific data series, and (*b*) indicates a treatment-specific data series. Otherwise, the *z*-score is 0.

Additionally, we took the gene families, GO terms (18), miRNA targets and pathway information from our functional annotations, described before as knowledge background sets. Users can submit their gene lists, derived from high-throughput experiments or other resources, and obtain the biological functions or molecular activities in which the submitted genes were significantly involved based on the p-values of statistical tests and the adjusted p-value after the false discovery rate correction (57).

$$P=\frac{\left(\frac{n}{k}\right)\left(\frac{N-n}{K-k}\right)}{\frac{N}{K}}$$

where *N* is the total number of sorghum genes (currently 33 032), and *n* is the number of genes in the query list. *K* represents the total number of genes in one gene set and *k* represents the number of overlapped genes. The default *P*-value is 0.05. 

## Visualization

Cytoscape (58) can be used to display the gene pairs by nodes and edges. We used different color and shape combinations to represent the nodes and edges. The yellow nodes indicate query genes or miRNAs, and the green nodes indicate the interaction elements. The orange lines link the positive co-expression pairs, while the blue color lines indicate negative co-expression pairs. To further enhance the links from gene nodes to each genes annotation, we added a pop-up window, which displays a short annotation when a gene from the network is clicked on (Figure 2E).

The datasets (GSE50464 and GSE54705) from GEO were changed from the sra to fastq format using the sratool (sratoolkit.2.4.0-1-centos_linux64). We used FastQC (fastqc_v0.10.1) to determine the data quality. Sequence reads were aligned to the sorghum genome through tophat (tophat-2.0.12.Linux_x86_64) and the annotation file was referred to using cufflinks (cufflinks-2.2.1.Linux_x86_64), which computed the fragments per kilobase of transcript per million mapped reads with the default parameters (59, 60) (Supplementary Table S5). The microarray dataset (GSE49879) probes corresponded to sorghum gene version 2.1 ID. After computing the RNA-seq data series and microarray data series, we calculated three expression matrices for different genotypes and tissues. To view each gene expression pattern, we used the open-flesh-chart (http://www.highcharts.com/) to draw the bar graph. The expression value was also included behind the bar graph (Figure 2C).

Gbrowse (v2.0) is a combination of databases and interactive web pages for manipulating and displaying annotations (like gene structure and location) on genomes. We have shown seven integrated genomic features, nuclear genome coding genes, miRNAs, ESTs, organelle genome information, repeat elements, mRNA-seq data and miRNA-seq data, in Gbrowse. Users can select the features that they are interested in, which will be displayed on the website (Supplementary Figure S1).

## Results

**Figure 1.**
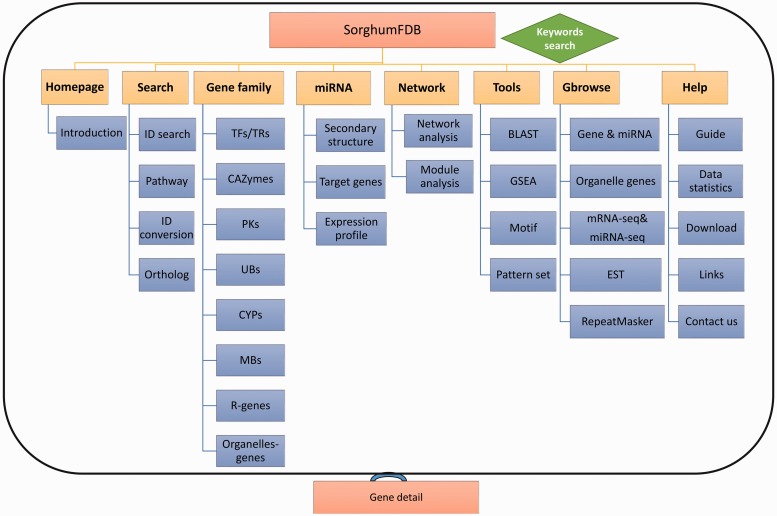
Overview of the sorghum database. The sorghum database has eight parts in total. The eight parts are shown in the picture as yellow rectangles, and the blue rectangles are the detailed classifications or annotations for the previous level. Finally, each part of the detailed information is linked to the gene annotation.

SorghumFDB was constructed under the LAMP (Linux + Apache + Mysql + PHP) environment. The dataset was uploaded to MySQL, and the web interface was built by PHP and Perl (http://www.perl.org) scripts. The SorghumFDB (Figure 1) has eight main parts. In the (a) ‘Search’ part, we have four special searching boxes, gene ID search, KEGG pathway, gene ID switch and ortholog search, as well as a keywords search function located on the navigation bar. The orthologous relationships define 111 461 pairs between 24 651 sorghum genes and 21 070 *Arabidopsis* genes, 19 691 pairs between 16 233 sorghum genes and 15 113 rice genes, and 21 176 pairs between 17 485 sorghum genes and 16 904 maize genes. The metabolic pathway includes 4399 genes in 131 KEGG pathways and 3377 genes in 535 PlantCyc (38) pathways. For the functional annotation, there are 3150 KOGs (17), 2836 Panthers (29), 32 796 Uniprot, 1177 GO (18) terms and 3832 domains. The (b) ‘Gene family’ part, contains eight commonly used families, 2541 TRs/TFs, 1253 PKs, 1166 CAZymes, 176 CYPs, 1846 UBs, 36 MBs, 231 R-genes and 194 organelle elements. The (c) ‘miRNA’ part has 242 miRNAs and 1742 predicted target genes. In addition, owing to the limited amount of research, some of the genes could not be annotated. Thus, we offer the (d) ‘Network’ part to identify genes that have similar functions as the query genes or related up-stream or down-stream genes. In conclusion, the multi-dimensional network contains 11 621 predicted PPIs with 3871 proteins, a co-expression network with 144 901 positive and 136 596 negative correlation pairs between 17 665 genes or miRNAs, and miRNA-target network with 4376 pairs. In other words, the network included 240 miRNAs and 21 494 genes, which covered 65.1% of the whole genome (33 032 genes). Then, the co-expression network was split into 987 modules containing 3954 genes by Cfinder (55). For the (e) ‘Tool’ part, four useful analysis tools, GSEA, motif analysis, BLAST and pattern set, that enabled a statistical analysis of differentially expressed genes (RNA-seq or microarray), modification-enriched (TF binding, histone modification and DNA methylation) genes and network genes, which could illustrate the related biological phenomena. In the (f) ‘Generic genome browser’ (Gbrowse) part, the locational information on the seven tracks, including sequence elements and high throughput data, are accessible (Table 1). All of the information was integrated into the gene or miRNA detail webpage. The bar graphs of expression profiles were also added to the detail pages. Additionally, the (g) ‘Homepage’ and (h) ‘Help’ webpages will help users to understand the key points, construction and accessibility of SorghumFDB.

**Figure 2.**
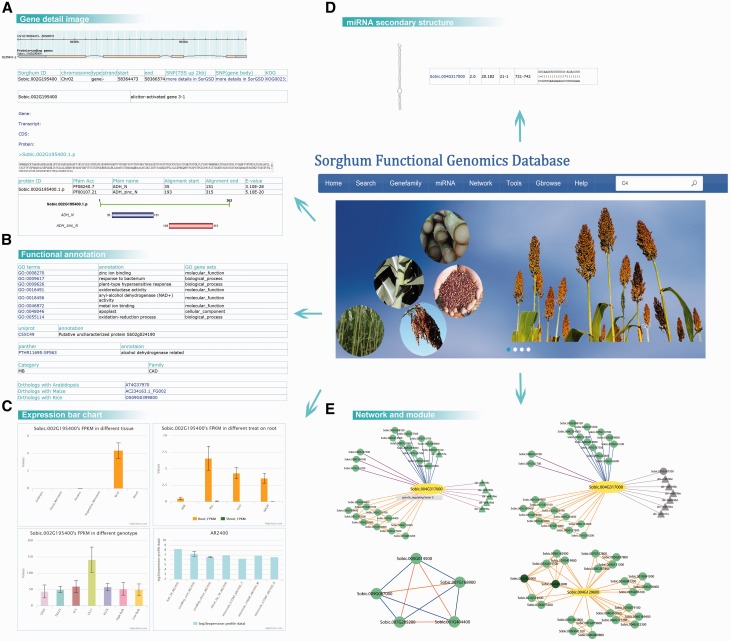
Detail information on the sorghum webpage. By searching with the gene *Sobic.002G195400*, we retrieved (A) the basic information on gene structure, location and sequence; (B) the GO, Uniprot, Panther and gene family annotations; (C) the expression patterns based on the data series GSE50464, GSE54705 and GSE49879, for different tissues, genotypes and treatments. (D) The secondary structure and complementary base pairing of miRNA sbi-miR396a. (E) The search results of *Sobic.004G317000*’s network and module 5.

We established some ways to introduce the uses of the SorghumFDB through the following: Sb02g024190 and Sb04g005950 (61) are two cinnamyl alcohol dehydrogenases (CADs), which are key enzymes in the lignin pathway that affect the biosynthesis of lignin monolignols (62). The search tool transformed the old version ID, *Sb02g024190*, to *Sobic.002G195400* (the sorghum version 2.1). After that, a gene details search displayed integrated information on the gene. For example, (i) the structure and location of the gene (Gbrowse); (ii) the gene, transcript, coding DNA sequence and protein sequence; (iii) the functional domain predicted by Pfam (37), such as the ‘ADH_N’ or ‘ADH_zinc_N’ domains (Figure 2A); (iv) the GO (18) term annotations, such as ‘zinc ion binding’ or ‘plant-type hypersensitive response’; (v) the Uniprot ID ‘C5XC49’ and Panther (29) ID ‘PTHR11695:SF563’; and the (vi) orthologous pairs in *Arabidopsis*, rice and maize (Figure 2B). In the bar graphs it was evident that the gene expression level was significantly high in roots in the data series of GSE50464 and was still expressed after the ABA treatment. However, in the data series of GSE54705, the genes were more highly expressed in the genotype of Ch17 than in other genotypes, such as CK60 and TX623 (Figure 2C).

Apart from the single gene annotation, searches for correlated relationships with other genes was provided. Here, we took *Sobic.004G317000*, a GRF transcript factor as an example. When we tried to analyse the network of the query gene, we could choose from several kinds of network, such as positive co-expression, negative co-expression, miRNA-targets and PPI networks (Figure 2E). To further analyse the expression specificity in different genotypes and tissues, we selected the sweet sorghum Atlas and stem to construct dynamic networks. In addition to the relationships with genes, relationships between functional modules could be displayed. For instance, *Sobic.004G129600* was not well annotated using other bioinformatics methods, but after the module analysis several overlapping modules contained it (Figure 2E). Thus, we could hypothesize the function of the query genes based on the modules.

In the gene *Sobic.004G317000*’s network, the pink line represented the gene targeted by the miRNA sbi-miR396 family. In addition, the miRNA detail webpage was included in the database. For example, the miRNA sbi-miR396a detail page displayed the miRNA sequence of mature, precursor and miRNA secondary structures, as well as the expression bar chart (Figure 2D).

Finally, some analysis tools have been added to our database, such as BLAST, gene set enrichment (57) and motif significance analyses, as well as a pattern set tool.

## Discussion

SorghumFDB contains single gene functional annotations and searches, as well as multiple gene analyses. Compared with SorghumFDB, multiple species synthesis databases, such as Phytozome and Gramene, may elaborate less data than that of single species functional genomics databases that possess analyses tools and gene family classifications involving species specificities and flexible updates. For the popular single species functional genomic databases in agricultural, such as TIGR, MaizeGDB and SIFGD, that integrates information from previous works. Except for basic functions that were referred to in other single species’ functional databases, we added new characteristics aimed at producing good agronomic traits and analyses tools.

SorghumFDB should be applied as a practical analysis tool. Biomass is derived from solar energy and a kind of renewable energy source. Lignocellulose biomass is the most important component. *S. bicolor* is a popular bioenergy plant that can relieve traditional fossil fuels shortages through the biosynthesis of cellulose. Sugarcane and corn, which are known to have a high biomass of sugar and starch, have become the first generation of biofuels, while sorghum and cotton became the new generation of bioenergy plants, owing to their abundant lignocellulosic biomass (63). Lignin is crucial for the structural integrity of the cell wall, and the stiffness and strength of the stem. However, a low lignin content is the prerequisite for enzymatic degradation of cellulose and hemicellulose, which can be further converted to the biofuel cellulosic ethanol. Nowadays, scientists dream of modifying lignin’s composition and content using bioengineering methods that improve the conversion efficiency from cellulose to ethyl alcohol (2).

**Figure 3.**
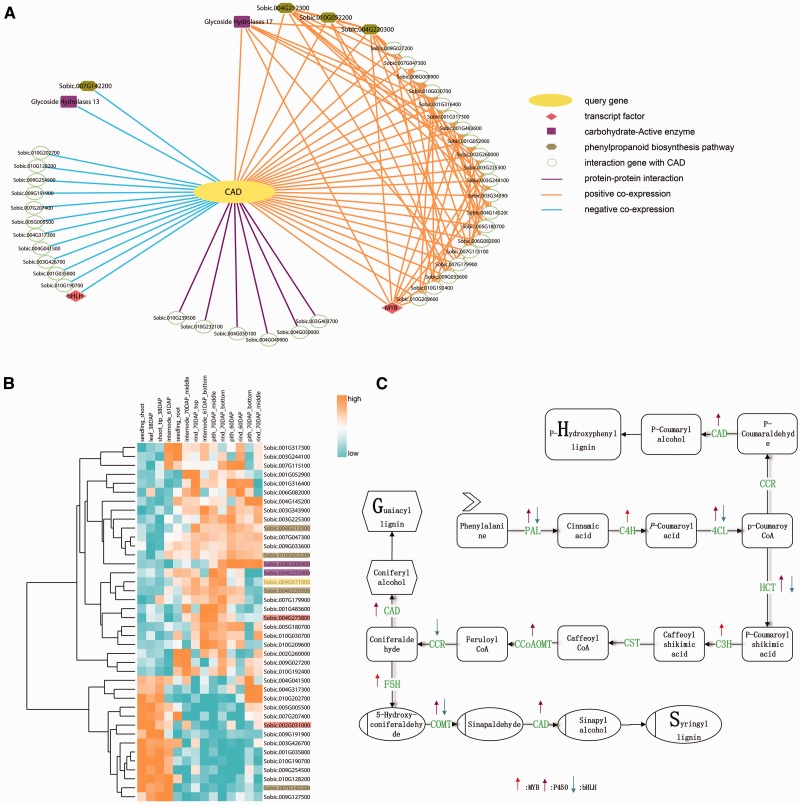
Network of the CAD gene *Sobic.004G071000*. **(**A) The CAD network. The query gene *Sobic.004G071000* is highlighted by yellow, the blue line represents the negative co-expression of the conjoined gene, the orange line represents the positive co-expression of the conjoined gene, and the purple line indicates that the two nodes have a PPI relationship. The pink rhombus represents TFs. Here, one is MYB and the other is bHLH. The purple rectangles represent the carbohydrate-active enzyme family. The dark yellow hexagonal gene participated in phenylpropanoid biosynthesis pathway. (B) The transcriptome analysis of all of the CAD co-expression network genes. We selected the genotype type of PI455230 and different tissue, such as seed and leaf, and used cluster 3.0 software to illustrate the expression profile. We also highlighted the important genes of the network with the same colors as in Figure 3A. (C) The lignin pathway illustrates the biosynthesis process of the three kinds of lignin monomers, S-, G- and H-lignin. In the pathway, the related regulation factors, as found in published papers, are labeled with arrows. The up arrows represent up-regulation, while the down arrows represent down-regulation.

Lignin is a kind of terrestrial biopolymer which rank only second to cellulose. Lignin is the product of phenylpropanoid pathway and contains primarily three types of subunits which are p-hydroxyphenyl (H), guaiacyl (G) and syringyl (S) lignin (6). Lignin biosynthesis pathway is mainly lignin monomer synthesis. P-coumaryl, coniferyl and sinapyl alcohols which experienced different degree of methoxylation (64, 65) are three dominant lignin monomers. After the synthesis of monolignol, they are transferred to deposition sites. In the end, through dehydrogenation and polymerization, they form lignin in the cell wall. The monolignol biosynthetic pathway was detailed introduced below in (Figure 3C) and the key enzymes were list in Supplementary Table S2 (6).

Another CAD gene (*Sobic.004G071000*) has two transcripts and the coding protein embed ‘ADH_N’ and ‘ADH_Zinc_N’ domains. At the same time, it was annotated by ‘alcohol dehydrogenase activity’, ‘zinc-dependent’ GO annotations and participated in phenylpropanoid biosynthesis pathway.

The network analysis was our starting point. The construction of a multidimensional network, as introduced above, was based on the microarray data. In the public NCBI platform, the transcriptome data, including microarray and RNA-seq, both had certain accumulation levels. While the differences in experimental designs, analyses processes and the normalization methods make it difficult to combine RNA-seq and microarray datasets. Transcriptome data covering broad biological samples could contribute to the accuracy of the constructed co-expression network. In particular, in large sample size research, microarray data was processed faster and easier, while the larger coverage of various conditions is necessary to detect subtle functional connections (53). The comparison of RNA-seq datasets, which focus on different treatments and tissues, with microarray datasets main results in research on diverse tissues and genotypes. The published database for sorghum transcriptomes, named MOROKOSHI, displays a network based on the RNA-seq dataset. Then, we used the microarray data to build a network. We analysed the CAD gene in both the internal network, which was computed to the microarray data (Figure 3A), and the outside network was based on the RNA-seq data. After a network analysis, the outside network found 19 genes with 23 positive co-expression relationships after transferring version 1 to version 2.1 and filtering for incomplete pairs (Supplementary Figure S5B). Additionally, SorghumFDB produced 45 genes that had relationships with the query genes, including 25 positive co-expressers, 14 negative co-expressers and 6 PPIs.

We applied all 46 genes (Table 2) in the selected network to a GSEA analysis using default parameters (Fisher’s exact test, *P*-value ≤ 0.05). These genes were significantly involved in lignin biosynthesis pathways, such as flavonoid and phenylpropanoid biosynthesis. Meanwhile, some of the key elements were found, including MYB, bHLH transcription factors and the carbohydrate-active enzyme family (Supplementary Table S3). These elements were previously reported to play important roles in the lignin pathway (62,65). To explain and highlight the key functions of the CAD network, we replaced some gene IDs with the corresponding annotation information from the result of the GSEA analysis using different colors and shapes as prescribed by the Cytoscape software (Figure 3A). For example, the query gene *Sobic.004G071000* was replaced with the CAD and highlighted in bright yellow. Meanwhile, the network genes from MOROKOSHI had two overlapping genes, except for the query gene, with our CAD network gene list (Supplementary Figure S5A). The specific genes (17) in the MOROKOSHI network had additional functions, such as CYP75A of the CYPs family, which has an unregulated function as seen in Figure 3C, which shows the GESA annotation analysis (66) (Supplementary Table S3). The co-expression network between the microarray and RNA-seq data may be complementary.

**Table 2.** Details of the CAD gene *Sobic.004G071000*’s network

Protein A	Protein B	PCC	MR	Relationship
*Sobic.004G273800*	*Sobic.004G071000*	0.74	20.49	Positive
*Sobic.001G316400*	*Sobic.004G071000*	0.74	9.38	Positive
*Sobic.002G260000*	*Sobic.004G071000*	0.77	6.928	Positive
*Sobic.007G115100*	*Sobic.004G071000*	0.82	4.898	Positive
*Sobic.003G225300*	*Sobic.004G071000*	0.81	4.898	Positive
*Sobic.005G180700*	*Sobic.004G071000*	0.77	20.39	Positive
*Sobic.003G244100*	*Sobic.004G071000*	0.73	12.72	Positive
*Sobic.003G343900*	*Sobic.004G071000*	0.74	19.74	Positive
*Sobic.004G145200*	*Sobic.004G071000*	0.75	19.49	Positive
*Sobic.004G071000*	*Sobic.004G220300*	0.83	4.898	Positive
*Sobic.004G071000*	*Sobic.001G317300*	0.69	28.61	Positive
*Sobic.004G071000*	*Sobic.001G483600*	0.79	4.898	Positive
*Sobic.004G071000*	*Sobic.010G052200*	0.79	22.44	Positive
*Sobic.004G071000*	*Sobic.009G033600*	0.77	27.14	Positive
*Sobic.004G071000*	*Sobic.010G192400*	0.7	28.46	Positive
*Sobic.004G071000*	*Sobic.007G047300*	0.84	3.162	Positive
*Sobic.004G071000*	*Sobic.010G209600*	0.77	13.49	Positive
*Sobic.004G071000*	*Sobic.009G027200*	0.67	15.87	Positive
*Sobic.004G071000*	*Sobic.007G179900*	0.8	7.071	Positive
*Sobic.004G071000*	*Sobic.008G008900*	0.73	28.98	Positive
*Sobic.004G071000*	*Sobic.006G082000*	0.73	12.04	Positive
*Sobic.004G071000*	*Sobic.004G212300*	0.78	17.6	Positive
*Sobic.004G071000*	*Sobic.001G052900*	0.8	2.449	Positive
*Sobic.004G071000*	*Sobic.004G232900*	0.79	3	Positive
*Sobic.004G071000*	*Sobic.010G030700*	0.77	5.656	Positive
*Sobic.004G273800*	*Sobic.001G316400*	0.72	17.54	Positive
*Sobic.004G273800*	*Sobic.003G225300*	0.76	18.33	Positive
*Sobic.004G273800*	*Sobic.003G343900*	0.73	21.63	Positive
*Sobic.004G273800*	*Sobic.004G220300*	0.75	24.49	Positive
*Sobic.004G273800*	*Sobic.001G483600*	0.8	2.828	Positive
*Sobic.004G273800*	*Sobic.010G209600*	0.75	14.96	Positive
*Sobic.004G273800*	*Sobic.007G179900*	0.84	1	Positive
*Sobic.004G273800*	*Sobic.004G232900*	0.74	9.165	Positive
*Sobic.001G316400*	*Sobic.003G225300*	0.74	8	Positive
*Sobic.001G316400*	*Sobic.004G220300*	0.77	5.196	Positive
*Sobic.001G316400*	*Sobic.001G483600*	0.71	22.97	Positive
*Sobic.001G316400*	*Sobic.007G047300*	0.74	16.88	Positive
*Sobic.001G316400*	*Sobic.004G212300*	0.73	22.8	Positive
*Sobic.001G316400*	*Sobic.004G232900*	0.69	27.92	Positive
*Sobic.002G260000*	*Sobic.003G244100*	0.7	12.84	Positive
*Sobic.002G260000*	*Sobic.004G145200*	0.78	4.242	Positive
*Sobic.002G260000*	*Sobic.001G483600*	0.74	8.124	Positive
*Sobic.002G260000*	*Sobic.010G192400*	0.81	1	Positive
*Sobic.002G260000*	*Sobic.009G027200*	0.64	25.9	Positive
*Sobic.002G260000*	*Sobic.004G232900*	0.72	8.366	Positive
*Sobic.002G260000*	*Sobic.010G030700*	0.72	11.48	Positive
*Sobic.007G115100*	*Sobic.003G225300*	0.77	29.34	Positive
*Sobic.007G115100*	*Sobic.003G244100*	0.73	20.97	Positive
*Sobic.007G115100*	*Sobic.010G052200*	0.81	22.58	Positive
*Sobic.007G115100*	*Sobic.009G033600*	0.79	28.58	Positive
*Sobic.007G115100*	*Sobic.007G047300*	0.81	15.71	Positive
*Sobic.007G115100*	*Sobic.007G179900*	0.81	9.899	Positive
*Sobic.007G115100*	*Sobic.001G052900*	0.76	18.43	Positive
*Sobic.003G225300*	*Sobic.005G180700*	0.84	1.414	Positive
*Sobic.003G225300*	*Sobic.003G343900*	0.78	10.9	Positive
*Sobic.003G225300*	*Sobic.004G220300*	0.79	15.49	Positive
*Sobic.003G225300*	*Sobic.001G483600*	0.82	2.236	Positive
*Sobic.003G225300*	*Sobic.007G047300*	0.83	6	Positive
*Sobic.003G225300*	*Sobic.010G209600*	0.8	6.928	Positive
*Sobic.003G225300*	*Sobic.004G212300*	0.78	22.64	Positive
*Sobic.003G225300*	*Sobic.010G030700*	0.77	5	Positive
*Sobic.005G180700*	*Sobic.003G244100*	0.72	23.83	Positive
*Sobic.005G180700*	*Sobic.004G220300*	0.82	8.366	Positive
*Sobic.005G180700*	*Sobic.007G047300*	0.79	23.66	Positive
*Sobic.005G180700*	*Sobic.010G209600*	0.84	2.449	Positive
*Sobic.003G244100*	*Sobic.007G047300*	0.75	13.26	Positive
*Sobic.003G244100*	*Sobic.004G212300*	0.72	27	Positive
*Sobic.003G244100*	*Sobic.010G030700*	0.69	17.97	Positive
*Sobic.003G343900*	*Sobic.004G145200*	0.73	26.26	Positive
*Sobic.003G343900*	*Sobic.001G483600*	0.77	8.366	Positive
*Sobic.003G343900*	*Sobic.007G047300*	0.83	3.741	Positive
*Sobic.003G343900*	*Sobic.004G212300*	0.76	22.24	Positive
*Sobic.004G145200*	*Sobic.010G192400*	0.76	8.062	Positive
*Sobic.004G145200*	*Sobic.007G047300*	0.81	6.782	Positive
*Sobic.004G145200*	*Sobic.008G008900*	0.73	29.98	Positive
*Sobic.004G220300*	*Sobic.010G052200*	0.87	4	Positive
*Sobic.004G220300*	*Sobic.007G047300*	0.84	7.416	Positive
*Sobic.004G220300*	*Sobic.007G179900*	0.82	6.708	Positive
*Sobic.004G220300*	*Sobic.004G212300*	0.85	4.898	Positive
*Sobic.004G220300*	*Sobic.004G232900*	0.71	27.92	Positive
*Sobic.001G317300*	*Sobic.006G082000*	0.67	29.98	Positive
*Sobic.001G317300*	*Sobic.010G030700*	0.69	15.87	Positive
*Sobic.001G483600*	*Sobic.010G209600*	0.77	9.165	Positive
*Sobic.001G483600*	*Sobic.004G232900*	0.75	4.898	Positive
*Sobic.001G483600*	*Sobic.010G030700*	0.75	7.937	Positive
*Sobic.010G052200*	*Sobic.009G033600*	0.89	4	Positive
*Sobic.010G052200*	*Sobic.007G047300*	0.91	2.828	Positive
*Sobic.010G052200*	*Sobic.004G212300*	0.85	10	Positive
*Sobic.009G033600*	*Sobic.007G047300*	0.91	1.732	Positive
*Sobic.009G033600*	*Sobic.004G212300*	0.8	21.02	Positive
*Sobic.010G192400*	*Sobic.009G027200*	0.66	14.56	Positive
*Sobic.010G192400*	*Sobic.010G030700*	0.75	6.708	Positive
*Sobic.007G047300*	*Sobic.004G212300*	0.92	1	Positive
*Sobic.010G209600*	*Sobic.010G030700*	0.75	10.95	Positive
*Sobic.009G027200*	*Sobic.001G052900*	0.73	3.741	Positive
*Sobic.007G179900*	*Sobic.004G232900*	0.73	18.33	Positive
*Sobic.008G008900*	*Sobic.006G082000*	0.74	8.66	Positive
*Sobic.006G082000*	*Sobic.001G052900*	0.74	6.633	Positive
*Sobic.007G207400*	*Sobic.004G071000*	−0.65	23.36	Negative
*Sobic.004G317300*	*Sobic.004G071000*	−0.7	17	Negative
*Sobic.002G031000*	*Sobic.004G071000*	−0.74	9.591	Negative
*Sobic.003G426700*	*Sobic.004G071000*	−0.74	21.09	Negative
*Sobic.004G071000*	*Sobic.005G005500*	−0.73	24.49	Negative
*Sobic.004G071000*	*Sobic.009G254500*	−0.73	10.58	Negative
*Sobic.004G071000*	*Sobic.001G035800*	−0.77	14.49	Negative
*Sobic.004G071000*	*Sobic.009G191900*	−0.72	29.25	Negative
*Sobic.004G071000*	*Sobic.004G041500*	−0.64	9.433	Negative
*Sobic.004G071000*	*Sobic.007G142200*	−0.71	14.69	Negative
*Sobic.004G071000*	*Sobic.010G128200*	−0.75	18.41	Negative
*Sobic.004G071000*	*Sobic.010G202700*	−0.7	9.165	Negative
*Sobic.004G071000*	*Sobic.010G190700*	−0.79	10.29	Negative
*Sobic.004G071000*	*Sobic.009G127500*	−0.7	14.14	Negative
*Sobic.004G071000*	*Sobic.010G232100*	–	–	Predicted PPI
*Sobic.004G071000*	*Sobic.003G403700*	–	–	Predicted PPI
*Sobic.004G071000*	*Sobic.004G049900*	–	–	Predicted PPI
*Sobic.004G071000*	*Sobic.004G050000*	–	–	Predicted PPI
*Sobic.004G071000*	*Sobic.004G050100*	–	–	Predicted PPI
*Sobic.004G071000*	*Sobic.010G239500*	–	–	Predicted PPI

Furthermore, we executed a motif enrichment analysis using the 46 genes. Among the significant enrichment (*P*-value < 0.05) motifs, some MYB- and bHLH-related binding sites were found. Notably, most of the network genes, including the query gene *Sobic.004G071000*, had MYB TF binding sites.

Then, a CAD co-expression network gene (GSE49879) expression matrix was used to investigate expression patterns. The data of PI455230, which contains most of the samples and tissues of the six genotypes, was used. A heatmap was produced by Cluster 3.0 software (67), with colors from blue to orange corresponding to expression profiles from low to high (Figure 3B). In the heatmap, we can see that *Sobic.004G071000* was highly expressed in rind and pind, instead of shoot and leaf. In addition, a similarity expression trend was seen in *Sobic.004G232900*, another lignin pathway-related gene. However, *Sobic.002G031000*, encoding a bHLH transcript factor, negatively regulated lignin biosynthesis, which had the opposite expression profile as the genes mentioned above.

Finally, lignin is mainly deposited on the stem, especially in more mature plants (68). Thus, we set an expression pattern threshold (high in the 70 DAP pith and rind, even in the 60 DAP pith and rind, and low in the last samples) to find more lignin pathway-related genes with the pattern set tool. As a result, we found 347 genes with a PCC threshold over 0.7, and the genes *Sobic.009G186600* (PCC = 0.76), *Sobic.010G245500* (PCC = 0.73) and *Sobic.009G186600* (PCC = 0.73), participate in the phenylalanine metabolism pathway (Supplementary Table S4).

Overall, our analyses results corroborated those of other published paper. For example, *Sobic.004G273800* is a MYB TF, and is up-regulated with the CAD gene (62), while *Sobic.002g031000* is a bHLH TF that is down-regulated with the CAD gene (65). In the network, genes also have been reported, such as *Sobic.004G220300* (phenylalanine ammonia lyase), *Sobic.010G052200* (Caffeoyl-CoA 3-O-methyltransferase, *Sobic.007G047300* (Caffeic acid O-methyltransferase) and *Sobic.004G212300* (Hydroxyl-cinnamoyl CoA:shikimate/quinate-Hydroxyl-cinnamoyltransferase) (16).

In a summary, we integrated analysis and visualization methods into SorghumFDB to predict the lignin pathway-related regulators and found genes that had been identified by published papers, as well as some new genes, that may play roles in metabolic processes. However, the analysis flow to integrate the network analysis, GSEA tool, pattern set and motif analysis, may have been a good way to explore the gene functions and regulatory relationship. In the development of sorghum research, newer annotations and tools may be added to our database to comprehensively analyse other biological problems, such as C4 photosynthesis and drought stress.

## Conclusion

At present, SorghumFDB is a platform for genome functional annotations and multi-dimensional network analyses. It encompassed most information, such as various annotations of whole genome assemblies, miRNA sequences and target genes, common gene families, network constructions using transcriptome data, PPI data and miRNA-target pairs, as well as multiple gene function annotation elements. Visualization tools (Gbrowse, Cytoscape and open-flash-chart) and four analysis-based tools, BLAST, GSEA, motif significance analysis and pattern set, were provided to determine the functional prediction. The SorghumFDB is online at http://structuralbiology.cau.edu.cn/sorghum/index.html. We hope that this will improve the accuracy and robustness of sorghum functional genomics analyses, and further help in understanding the gene regulatory networks involved in effective crop improvement.

## Supplementary data

Supplementary data are available at *Database* Online. 

## Funding

This work was supported by grants from the Ministry of Science and Technology of China (31371291 and 2012CB215301).

*Conflict of interest*. None declared.

## Supplementary Material

Supplementary Data
