# Supplementary material for: SorghumFDB: sorghum functional genomics database with multidimensional network analysis
Source: Database (Oxford). 2016 Jun 27;2016:baw099. doi: 10.1093/database/baw099 (PMC4921789; doi:10.1093/database/baw099)
Supplement: Supplementary Data [file supp_2016_baw099_index.html]

Supplementary Data 

# SorghumFDB: sorghum functional genomics database with multidimensional network analysis

## Supplementary Data

files

- Supplementary Data - zip file
