# Supplementary figures and images for: SorghumFDB: sorghum functional genomics database with multidimensional network analysis
Source: Database (Oxford). 2016 Jun 27;2016:baw099. doi: 10.1093/database/baw099 (PMC4921789; doi:10.1093/database/baw099)

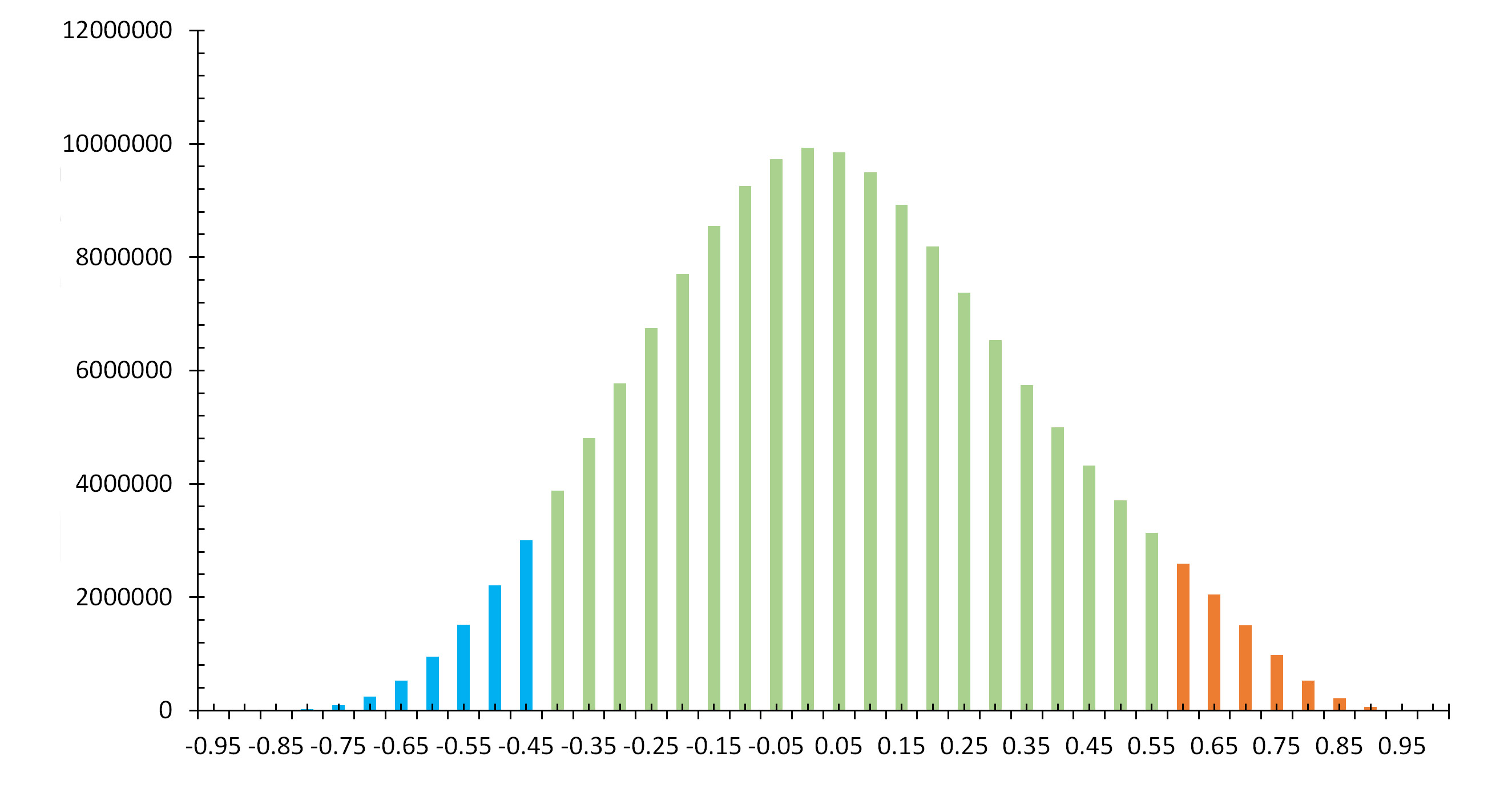

Supplement: Supplementary Data [file supp_baw099_suppl_data.zip › Figure S2.jpg]

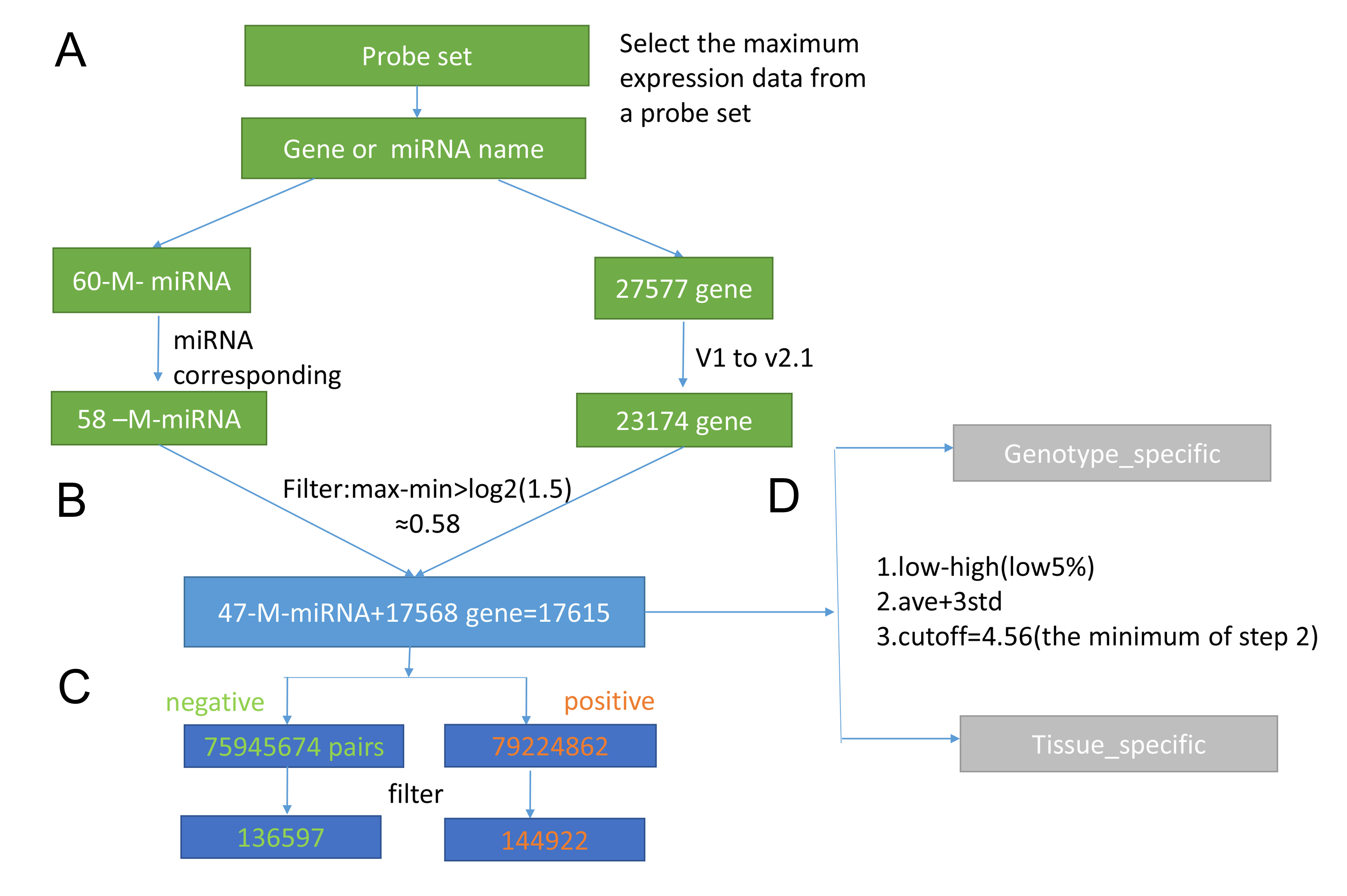

Supplement: Supplementary Data [file supp_baw099_suppl_data.zip › Figure S3.jpg]

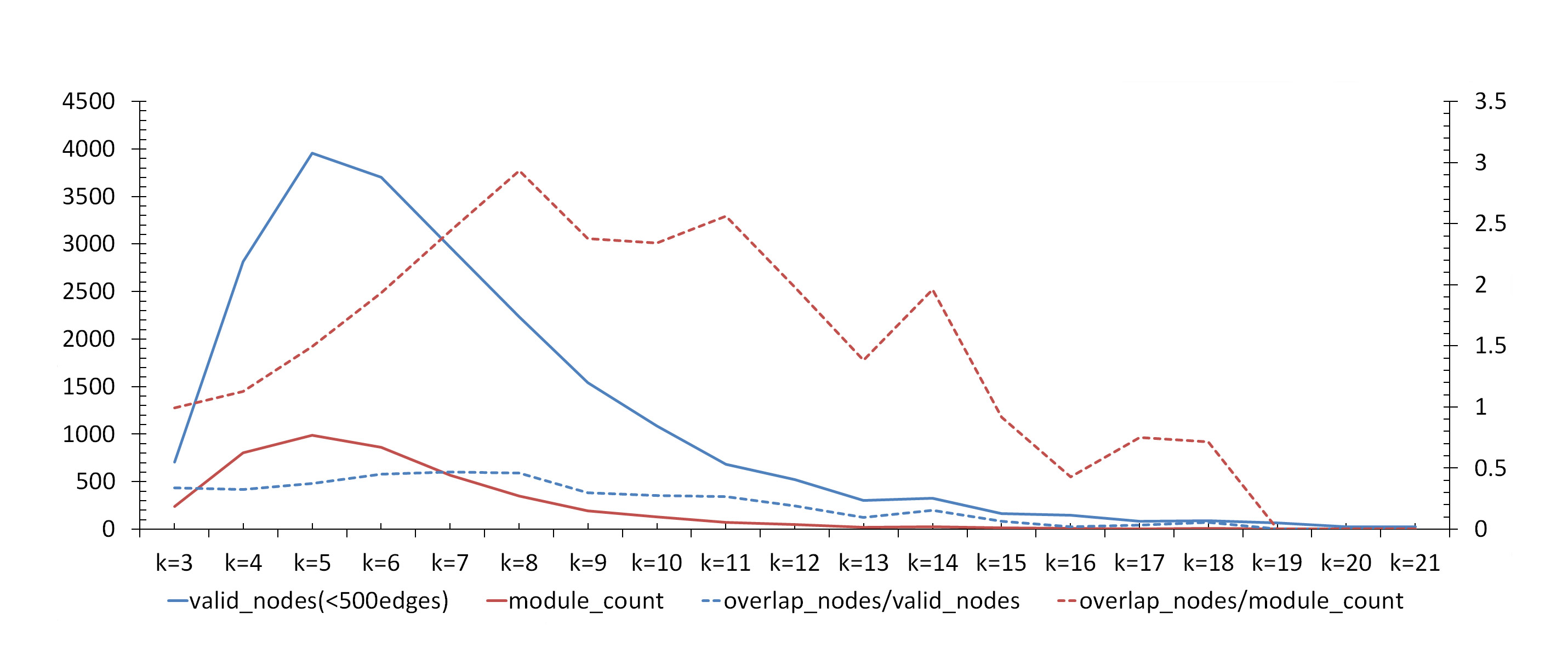

Supplement: Supplementary Data [file supp_baw099_suppl_data.zip › Figure S4.jpg]

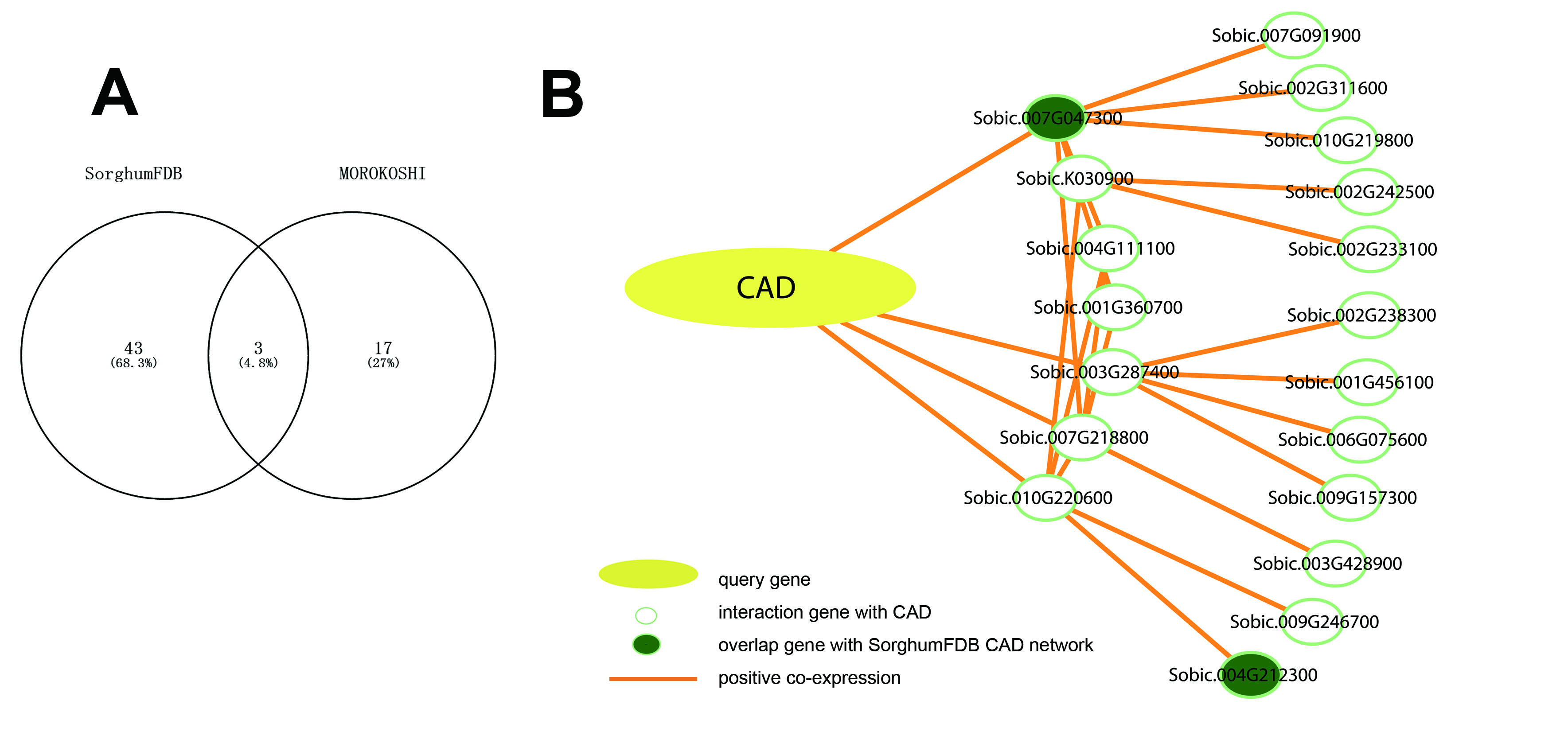

Supplement: Supplementary Data [file supp_baw099_suppl_data.zip › Figure S5.jpg]

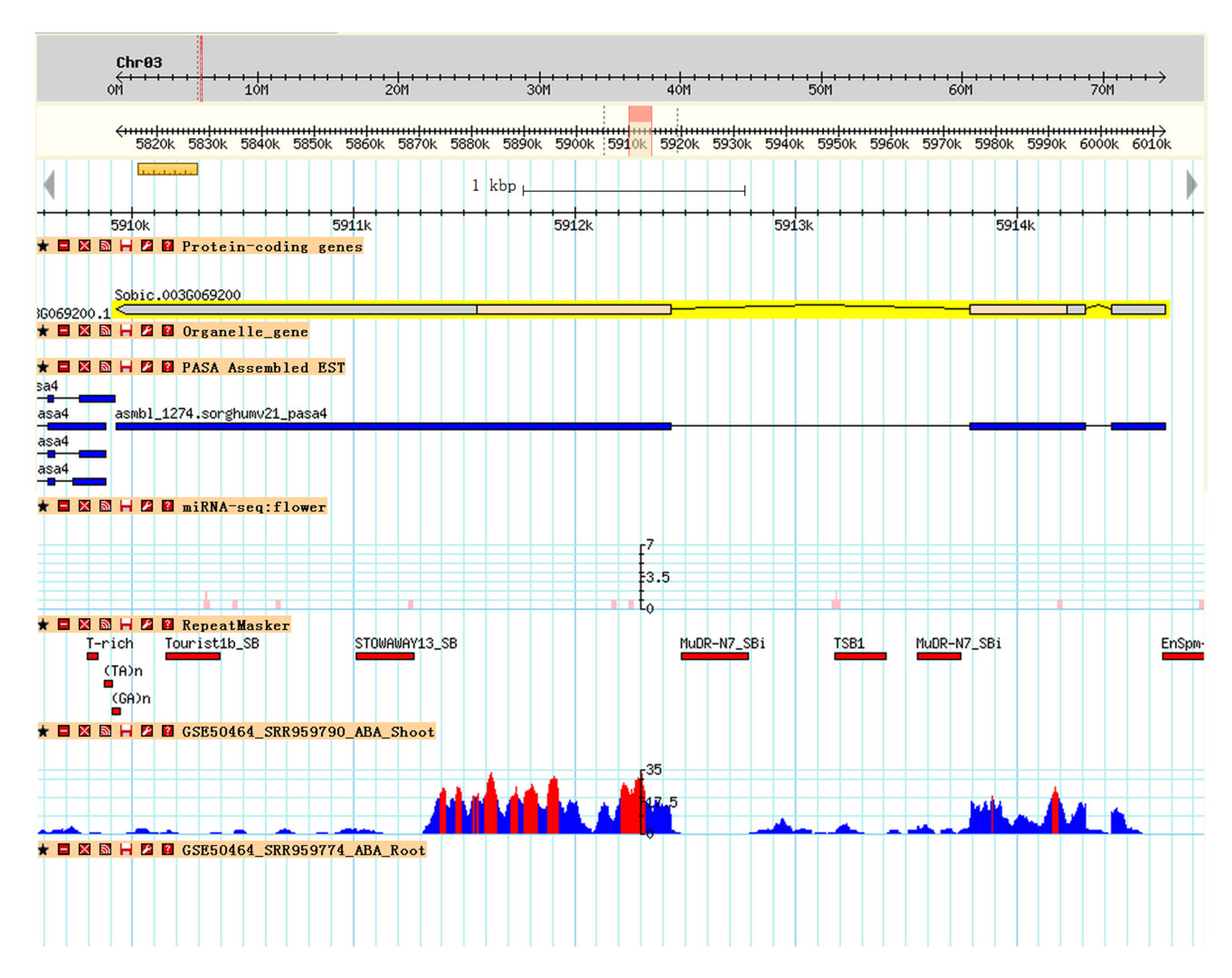

Supplement: Supplementary Data [file supp_baw099_suppl_data.zip › Figure S1.jpg]
