# Supplementary material for: SorghumFDB: sorghum functional genomics database with multidimensional network analysis
Source: Database (Oxford). 2016 Jun 27;2016:baw099. doi: 10.1093/database/baw099 (PMC4921789; doi:10.1093/database/baw099)
Supplement: Supplementary Data [file supp_baw099_suppl_data.zip › Supp_Tbls_2016-5-4.doc]

**Supplementary Table S1.** GSE49879 data series.

| Samples | Description |
| --- | --- |
| GSM1208512 | seedling_shoot_PI455230_1 |
| GSM1208513 | seedling_shoot_PI455230_2 |
| GSM1208514 | seedling_root_PI455230 |
| GSM1208515 | leaf_38DAP_ PI455230 |
| GSM1208516 | shoot_tip_38DAP_ PI455230 |
| GSM1208517 | internode_61DAP_PI455230 |
| GSM1208518 | rind_70DAP_PI455230_T |
| GSM1208519 | seedling_shoot_R159_1 |
| GSM1208520 | seedling_shoot_R159_2 |
| GSM1208521 | seedling_root_R159 |
| GSM1208522 | leaf_38DAP_ R159 |
| GSM1208523 | shoot_tip_38DAP_ R159 |
| GSM1208524 | internode_61DAP_R159_T |
| GSM1208525 | pith_70DAP_R159_T |
| GSM1208526 | rind_70DAP_R159_T |
| GSM1208527 | internode_70DAP_PI455230_M |
| GSM1208528 | internode_61DAP_PI455230_B |
| GSM1208529 | pith_70DAP_PI455230_M |
| GSM1208530 | pith_70DAP_PI455230_B |
| GSM1208531 | rind_70DAP_PI455230_M |
| GSM1208532 | rind_70DAP_PI455230_B |
| GSM1208533 | pith_60DAP_PI455230_1 |
| GSM1208534 | pith_60DAP_PI455230_2 |
| GSM1208535 | pith_60DAP_PI455230_3 |
| GSM1208536 | rind_60DAP_PI455230_1 |
| GSM1208537 | rind_60DAP_PI455230_2 |
| GSM1208538 | rind_60DAP_PI455230_3 |
| GSM1208539 | internode_61DAP_R159_B |
| GSM1208540 | pith_70DAP_R159_B |
| GSM1208541 | rind_70DAP_R159_B |
| GSM1208542 | pith_60DAP_R159 |
| GSM1208543 | rind_60DAP_R159 |
| GSM1208544 | internode_61DAP_Atlas_T |
| GSM1208545 | internode_70DAP_Atlas_M |
| GSM1208546 | internode_61DAP_Atlas_B |
| GSM1208547 | seedling_shoot_Atlas_1 |
| GSM1208548 | seedling_shoot_Atlas_2 |
| GSM1208549 | seedling_root_Atlas |
| GSM1208550 | leaf_38_Atlas |
| GSM1208551 | shoot_tip_38_Atlas |
| GSM1208552 | pith_70DAP_Atlas_T |
| GSM1208553 | pith_70DAP_Atlas_M |
| GSM1208554 | pith_70DAP_Atlas_B |
| GSM1208555 | rind_70DAP_Atlas_T |
| GSM1208556 | rind_70DAP_Atlas_M |
| GSM1208557 | rind_70DAP_Atlas_B |
| GSM1208558 | pith_60DAP_Atlas_1 |
| GSM1208559 | pith_60DAP_Atlas_2 |
| GSM1208560 | pith_60DAP_Atlas_3 |
| GSM1208561 | rind_60DAP_Atlas_1 |
| GSM1208562 | rind_60DAP_Atlas_2 |
| GSM1208563 | rind_60DAP_Atlas_3 |
| GSM1208564 | internode_61DAP_PI152611_T |
| GSM1208565 | internode_70DAP_PI152611_M |
| GSM1208566 | internode_61DAP_PI152611_B |
| GSM1208567 | seedling_shoot_PI152611_1 |
| GSM1208568 | seedling_shoot_PI152611_2 |
| GSM1208569 | seedling_root_PI152611 |
| GSM1208570 | leaf_38_PI152611 |
| GSM1208571 | shoot_tip_38_PI152611 |
| GSM1208572 | internode_61DAP_AR2400_T |
| GSM1208573 | internode_70DAP_AR2400_M |
| GSM1208574 | internode_61DAP_AR2400_B |
| GSM1208575 | seedling_shoot_AR2400_1 |
| GSM1208576 | seedling_shoot_AR2400_2 |
| GSM1208577 | seedling_root_AR2400_1 |
| GSM1208578 | seedling_root_AR2400_2 |
| GSM1208579 | leaf_38_AR2400 |
| GSM1208580 | shoot_tip_38_AR2400 |
| GSM1208581 | internode_61DAP_Fremont_T |
| GSM1208582 | internode_70DAP_Fremont_M |
| GSM1208583 | internode_61DAP_Fremont_B |
| GSM1208584 | seedling_shoot_Fremont_1 |
| GSM1208585 | seedling_shoot_Fremont_2 |
| GSM1208586 | seedling_root_Fremont_1 |
| GSM1208587 | seedling_root_Fremont_2 |
| GSM1208588 | leaf_38_Fremont |
| GSM1208589 | shoot_tip_38_Fremont |

**Supplementary** **table S2.** Lignin pathway key enzymes.

| Abbreviation | Enzyme full name |
| --- | --- |
| PAL | phenylalanine ammonia-lyase |
| 4CL | 4-coumarate:CoA ligase |
| C4H | cinnamate 4-hydroxylase |
| HCT | hydroxycinnamoyl CoA:shikimate hydroxycinnamoyl transferase |
| C3H | coumaroyl shikimate 3’-hydroxylase |
| CCoAOMT | caffeoyl-CoA 3-O-methyltransferase |
| CCR | cinnamoyl CoA reductases |
| F5H | ferulate 5-hydroxylase |
| COMT | caffeic acid 3-Omethyltransferase |
| CAD | cinnamyl alcohol dehydrogenase |

**Supplementary Table S3.** The CAD network gene function enrichment results.

| Source | Category | Description | NO. Genes | p value | FDR |
| --- | --- | --- | --- | --- | --- |
| SorghumFDB | ubiquitin_family | UBQ-PIM | 2 | 4.70E-04 | 0.0234 |
| SorghumFDB | monolignol_biosynthesis_family | CCoAOMT,Caffeoyl-CoA_8-O-methyltransferase | 1 | 2.78E-03 | 0.0346 |
| SorghumFDB | monolignol_biosynthesis_family | COMT,Caffeic_acid_O-methyltransferase | 1 | 2.78E-03 | 0.0346 |
| SorghumFDB | monolignol_biosynthesis_family | HCT,Hydroxyl-cinnamoyl_CoA:shikimate/quinate-Hydroxyl-cinnamoyltransferase | 1 | 2.78E-03 | 0.0346 |
| SorghumFDB | biological_process | GO:0007568 aging | 6 | 2.23E-11 | 6.27E-09 |
| SorghumFDB | biological_process | GO:0006464 cellular_protein_modification_process | 6 | 8.85E-11 | 1.24E-08 |
| SorghumFDB | biological_process | GO:0009751 response_to_salicylic_acid | 6 | 2.94E-09 | 2.75E-07 |
| SorghumFDB | biological_process | GO:0009809 lignin_biosynthetic_process | 4 | 1.30E-07 | 9.13E-06 |
| SorghumFDB | biological_process | GO:0006511 ubiquitin-dependent_protein_catabolic_process | 6 | 3.37E-07 | 1.89E-05 |
| SorghumFDB | cellular_component | GO:0005730 nucleolus | 7 | 1.10E-06 | 6.92E-05 |
| SorghumFDB | cellular_component | GO:0005618 cell_wall | 7 | 6.91E-06 | 1.45E-04 |
| SorghumFDB | cellular_component | GO:0005774 vacuolar_membrane | 6 | 5.68E-06 | 1.45E-04 |
| SorghumFDB | cellular_component | GO:0005773 vacuole | 6 | 1.90E-04 | 2.99E-03 |
| SorghumFDB | cellular_component | GO:0005622 intracellular | 6 | 6.66E-04 | 8.38E-03 |
| SorghumFDB | biological_process | GO:0009963 positive_regulation_of_flavonoid_biosynthetic_process | 2 | 1.96E-04 | 9.19E-03 |
| SorghumFDB | biological_process | GO:0009698 phenylpropanoid_metabolic_process | 2 | 4.29E-04 | 0.0172 |
| SorghumFDB | pathway | stilbenoid, diarylheptanoid_and_gingerol_biosynthesis | 3 | 5.67E-06 | 2.58E-04 |
| SorghumFDB | pathway | suberin_monomers_biosynthesis | 3 | 4.43E-05 | 9.64E-04 |
| SorghumFDB | pathway | flavonoid_biosynthesis | 3 | 6.63E-05 | 1.51E-03 |
| SorghumFDB | pathway | superpathway_of_scopolin_and_esculin_biosynthesis | 2 | 4.70E-04 | 5.11E-03 |
| SorghumFDB | pathway | phenylpropanoid_biosynthesis | 4 | 3.58E-04 | 5.44E-03 |
| MOROKOSHI | monolignol_biosynthesis_family | CCoAOMT,Caffeoyl-CoA_7-O-methyltransferase | 1 | 1.03E-03 | 0.0226 |
| MOROKOSHI | biological_process | GO:0010119 regulation_of_stomatal_movement | 2 | 3.64E-04 | 0.0347 |
| MOROKOSHI | cytochrome_p450_family | CYP75A | 1 | 1.54E-03 | 0.0226 |
| MOROKOSHI | pathway | suberin_monomers_biosynthesis | 1 | 2.39E-02 | 0.0239 |

**Supplementary table S4.** Pattern set results for the expression (high in the 70 DAP pith and rind, even in the 6 0DAP pith and rind, and low in the last samples).

| Gene | Correction |
| --- | --- |
| *Sobic.004G167500* | 0.88 |
| *Sobic.001G481600* | 0.88 |
| *Sobic.008G151300* | 0.87 |
| *Sobic.002G412800* | 0.87 |
| *Sobic.010G171300* | 0.86 |
| *Sobic.005G019800* | 0.86 |
| *Sobic.006G246500* | 0.85 |
| *Sobic.006G087900* | 0.85 |
| *Sobic.004G174600* | 0.85 |
| *Sobic.001G077700* | 0.84 |
| *Sobic.009G215100* | 0.83 |
| *Sobic.007G146800* | 0.83 |
| *Sobic.003G374600* | 0.83 |
| *Sobic.002G148700* | 0.83 |
| *Sobic.001G479800* | 0.83 |
| *Sobic.007G056900* | 0.82 |
| *Sobic.007G054500* | 0.82 |
| *Sobic.006G230800* | 0.82 |
| *Sobic.003G065100* | 0.82 |
| *Sobic.001G107800* | 0.82 |
| *Sobic.010G088600* | 0.81 |
| *Sobic.006G230900* | 0.81 |
| *Sobic.006G104600* | 0.81 |
| *Sobic.005G230000* | 0.81 |
| *Sobic.004G331800* | 0.81 |
| *Sobic.004G202700* | 0.81 |
| *Sobic.004G036400* | 0.81 |
| *Sobic.003G248400* | 0.81 |
| *Sobic.003G044900* | 0.81 |
| *Sobic.002G430500* | 0.81 |
| *Sobic.002G395600* | 0.81 |
| *Sobic.002G365400* | 0.81 |
| *Sobic.002G322900* | 0.81 |
| *Sobic.002G192000* | 0.81 |
| *Sobic.002G176000* | 0.81 |
| *Sobic.001G108900* | 0.81 |
| *Sobic.001G069200* | 0.81 |
| *Sobic.009G153600* | 0.8 |
| *Sobic.008G056000* | 0.8 |
| *Sobic.008G036900* | 0.8 |
| *Sobic.007G157700* | 0.8 |
| *Sobic.005G121300* | 0.8 |
| *Sobic.004G300300* | 0.8 |
| *Sobic.002G212000* | 0.8 |
| *Sobic.002G042000* | 0.8 |
| *Sobic.001G507600* | 0.8 |
| *Sobic.001G028800* | 0.8 |
| *Sobic.009G199400* | 0.79 |
| *Sobic.009G032500* | 0.79 |
| *Sobic.008G090600* | 0.79 |
| *Sobic.007G055500* | 0.79 |
| *Sobic.006G069900* | 0.79 |
| *Sobic.004G350000* | 0.79 |
| *Sobic.004G267400* | 0.79 |
| *Sobic.003G292000* | 0.79 |
| *Sobic.003G199500* | 0.79 |
| *Sobic.003G176700* | 0.79 |
| *Sobic.003G076600* | 0.79 |
| *Sobic.002G295400* | 0.79 |
| *Sobic.002G043400* | 0.79 |
| *Sobic.001G494700* | 0.79 |
| *Sobic.010G248100* | 0.78 |
| *Sobic.010G210700* | 0.78 |
| *Sobic.008G072900* | 0.78 |
| *Sobic.007G126200* | 0.78 |
| *Sobic.006G280300* | 0.78 |
| *Sobic.006G222300* | 0.78 |
| *Sobic.006G098200* | 0.78 |
| *Sobic.006G003600* | 0.78 |
| *Sobic.005G043100* | 0.78 |
| *Sobic.004G327800* | 0.78 |
| *Sobic.004G305600* | 0.78 |
| *Sobic.004G262600* | 0.78 |
| *Sobic.004G069700* | 0.78 |
| *Sobic.003G246100* | 0.78 |
| *Sobic.003G148300* | 0.78 |
| *Sobic.002G422400* | 0.78 |
| *Sobic.002G372300* | 0.78 |
| *Sobic.002G366500* | 0.78 |
| *Sobic.001G543900* | 0.78 |
| *Sobic.001G004000* | 0.78 |
| *Sobic.010G059800* | 0.77 |
| *Sobic.009G230700* | 0.77 |
| *Sobic.009G160800* | 0.77 |
| *Sobic.009G143400* | 0.77 |
| *Sobic.007G045500* | 0.77 |
| *Sobic.007G040500* | 0.77 |
| *Sobic.005G044800* | 0.77 |
| *Sobic.004G349100* | 0.77 |
| *Sobic.004G304900* | 0.77 |
| *Sobic.004G209000* | 0.77 |
| *Sobic.004G079100* | 0.77 |
| *Sobic.004G058000* | 0.77 |
| *Sobic.003G362700* | 0.77 |
| *Sobic.003G115300* | 0.77 |
| *Sobic.002G405900* | 0.77 |
| *Sobic.002G218800* | 0.77 |
| *Sobic.002G211500* | 0.77 |
| *Sobic.002G205100* | 0.77 |
| *Sobic.002G118700* | 0.77 |
| *Sobic.002G012200* | 0.77 |
| *Sobic.001G388300* | 0.77 |
| *Sobic.001G311500* | 0.77 |
| *Sobic.001G113700* | 0.77 |
| *Sobic.010G247300* | 0.76 |
| *Sobic.010G245400* | 0.76 |
| *Sobic.009G227900* | 0.76 |
| *Sobic.007G188800* | 0.76 |
| *Sobic.006G183200* | 0.76 |
| *Sobic.006G045000* | 0.76 |
| *Sobic.005G116300* | 0.76 |
| *Sobic.005G019600* | 0.76 |
| *Sobic.004G313000* | 0.76 |
| *Sobic.004G299900* | 0.76 |
| *Sobic.004G256800* | 0.76 |
| *Sobic.004G188300* | 0.76 |
| *Sobic.004G146800* | 0.76 |
| *Sobic.004G085900* | 0.76 |
| *Sobic.004G059400* | 0.76 |
| *Sobic.003G442900* | 0.76 |
| *Sobic.003G376000* | 0.76 |
| *Sobic.003G186500* | 0.76 |
| *Sobic.002G372100* | 0.76 |
| *Sobic.002G362000* | 0.76 |
| *Sobic.001G478800* | 0.76 |
| *Sobic.001G448800* | 0.76 |
| *Sobic.001G440600* | 0.76 |
| *Sobic.001G419100* | 0.76 |
| *Sobic.001G383000* | 0.76 |
| *Sobic.001G314800* | 0.76 |
| *Sobic.001G259100* | 0.76 |
| *Sobic.001G234300* | 0.76 |
| *Sobic.001G185400* | 0.76 |
| *Sobic.001G092600* | 0.76 |
| *Sobic.010G250600* | 0.75 |
| *Sobic.010G104100* | 0.75 |
| *Sobic.010G050900* | 0.75 |
| *Sobic.009G258300* | 0.75 |
| *Sobic.009G065000* | 0.75 |
| *Sobic.008G097400* | 0.75 |
| *Sobic.008G059400* | 0.75 |
| *Sobic.007G223200* | 0.75 |
| *Sobic.007G092900* | 0.75 |
| *Sobic.006G231400* | 0.75 |
| *Sobic.006G193900* | 0.75 |
| *Sobic.006G174900* | 0.75 |
| *Sobic.005G116900* | 0.75 |
| *Sobic.005G026100* | 0.75 |
| *Sobic.004G325400* | 0.75 |
| *Sobic.003G386700* | 0.75 |
| *Sobic.003G318700* | 0.75 |
| *Sobic.003G310700* | 0.75 |
| *Sobic.003G238100* | 0.75 |
| *Sobic.003G056500* | 0.75 |
| *Sobic.002G203100* | 0.75 |
| *Sobic.002G182800* | 0.75 |
| *Sobic.001G217300* | 0.75 |
| *Sobic.001G039800* | 0.75 |
| *Sobic.K041000* | 0.74 |
| *Sobic.010G267700* | 0.74 |
| *Sobic.010G089400* | 0.74 |
| *Sobic.010G034800* | 0.74 |
| *Sobic.009G244700* | 0.74 |
| *Sobic.009G111200* | 0.74 |
| *Sobic.009G023200* | 0.74 |
| *Sobic.008G109900* | 0.74 |
| *Sobic.008G020800* | 0.74 |
| *Sobic.007G202900* | 0.74 |
| *Sobic.007G038500* | 0.74 |
| *Sobic.005G167600* | 0.74 |
| *Sobic.004G301900* | 0.74 |
| *Sobic.004G271200* | 0.74 |
| *Sobic.004G059900* | 0.74 |
| *Sobic.004G014600* | 0.74 |
| *Sobic.003G424500* | 0.74 |
| *Sobic.003G400100* | 0.74 |
| *Sobic.003G399900* | 0.74 |
| *Sobic.003G367300* | 0.74 |
| *Sobic.003G342600* | 0.74 |
| *Sobic.003G288300* | 0.74 |
| *Sobic.003G279300* | 0.74 |
| *Sobic.003G087600* | 0.74 |
| *Sobic.003G034700* | 0.74 |
| *Sobic.002G431100* | 0.74 |
| *Sobic.002G401600* | 0.74 |
| *Sobic.002G389200* | 0.74 |
| *Sobic.002G331500* | 0.74 |
| *Sobic.002G173800* | 0.74 |
| *Sobic.002G166800* | 0.74 |
| *Sobic.002G132000* | 0.74 |
| *Sobic.002G125900* | 0.74 |
| *Sobic.001G531700* | 0.74 |
| *Sobic.001G479300* | 0.74 |
| *Sobic.001G423100* | 0.74 |
| *Sobic.001G258500* | 0.74 |
| *Sobic.001G200800* | 0.74 |
| *Sobic.K019600* | 0.73 |
| *Sobic.010G245500* | 0.73 |
| *Sobic.010G203200* | 0.73 |
| *Sobic.010G194900* | 0.73 |
| *Sobic.010G194100* | 0.73 |
| *Sobic.010G126400* | 0.73 |
| *Sobic.009G186600* | 0.73 |
| *Sobic.009G161700* | 0.73 |
| *Sobic.009G155500* | 0.73 |
| *Sobic.009G135900* | 0.73 |
| *Sobic.009G046600* | 0.73 |
| *Sobic.009G002000* | 0.73 |
| *Sobic.008G124400* | 0.73 |
| *Sobic.008G003100* | 0.73 |
| *Sobic.006G254700* | 0.73 |
| *Sobic.006G176100* | 0.73 |
| *Sobic.006G128900* | 0.73 |
| *Sobic.005G121200* | 0.73 |
| *Sobic.005G104900* | 0.73 |
| *Sobic.005G084900* | 0.73 |
| *Sobic.005G049000* | 0.73 |
| *Sobic.004G204100* | 0.73 |
| *Sobic.004G166000* | 0.73 |
| *Sobic.003G431000* | 0.73 |
| *Sobic.003G428900* | 0.73 |
| *Sobic.003G425200* | 0.73 |
| *Sobic.003G309900* | 0.73 |
| *Sobic.003G293500* | 0.73 |
| *Sobic.003G275100* | 0.73 |
| *Sobic.003G058400* | 0.73 |
| *Sobic.003G055700* | 0.73 |
| *Sobic.003G000400* | 0.73 |
| *Sobic.002G330700* | 0.73 |
| *Sobic.002G245800* | 0.73 |
| *Sobic.002G207500* | 0.73 |
| *Sobic.002G166700* | 0.73 |
| *Sobic.002G166600* | 0.73 |
| *Sobic.002G165700* | 0.73 |
| *Sobic.002G141000* | 0.73 |
| *Sobic.002G043500* | 0.73 |
| *Sobic.002G020900* | 0.73 |
| *Sobic.002G012000* | 0.73 |
| *Sobic.002G009900* | 0.73 |
| *Sobic.001G514000* | 0.73 |
| *Sobic.001G403300* | 0.73 |
| *Sobic.001G389700* | 0.73 |
| *Sobic.001G382000* | 0.73 |
| *Sobic.001G196400* | 0.73 |
| *Sobic.001G076400* | 0.73 |
| *Sobic.001G069400* | 0.73 |
| *Sobic.010G121000* | 0.72 |
| *Sobic.010G071400* | 0.72 |
| *Sobic.010G063200* | 0.72 |
| *Sobic.009G218800* | 0.72 |
| *Sobic.009G186300* | 0.72 |
| *Sobic.009G147200* | 0.72 |
| *Sobic.009G073100* | 0.72 |
| *Sobic.008G043500* | 0.72 |
| *Sobic.008G038100* | 0.72 |
| *Sobic.007G192400* | 0.72 |
| *Sobic.007G094600* | 0.72 |
| *Sobic.006G240500* | 0.72 |
| *Sobic.005G181000* | 0.72 |
| *Sobic.004G237400* | 0.72 |
| *Sobic.004G227400* | 0.72 |
| *Sobic.004G190100* | 0.72 |
| *Sobic.004G102800* | 0.72 |
| *Sobic.003G396300* | 0.72 |
| *Sobic.003G391900* | 0.72 |
| *Sobic.003G350700* | 0.72 |
| *Sobic.003G278500* | 0.72 |
| *Sobic.003G159600* | 0.72 |
| *Sobic.002G289900* | 0.72 |
| *Sobic.002G288400* | 0.72 |
| *Sobic.002G212200* | 0.72 |
| *Sobic.001G510700* | 0.72 |
| *Sobic.001G435800* | 0.72 |
| *Sobic.001G342400* | 0.72 |
| *Sobic.001G289800* | 0.72 |
| *Sobic.001G287100* | 0.72 |
| *Sobic.001G266400* | 0.72 |
| *Sobic.001G031200* | 0.72 |
| *Sobic.010G265600* | 0.71 |
| *Sobic.010G223600* | 0.71 |
| *Sobic.010G084100* | 0.71 |
| *Sobic.009G136300* | 0.71 |
| *Sobic.008G046600* | 0.71 |
| *Sobic.008G009900* | 0.71 |
| *Sobic.007G221000* | 0.71 |
| *Sobic.007G212200* | 0.71 |
| *Sobic.007G168500* | 0.71 |
| *Sobic.007G072000* | 0.71 |
| *Sobic.007G068800* | 0.71 |
| *Sobic.006G279700* | 0.71 |
| *Sobic.006G207500* | 0.71 |
| *Sobic.006G194300* | 0.71 |
| *Sobic.006G077900* | 0.71 |
| *Sobic.005G224700* | 0.71 |
| *Sobic.005G045900* | 0.71 |
| *Sobic.005G043400* | 0.71 |
| *Sobic.004G325700* | 0.71 |
| *Sobic.004G250900* | 0.71 |
| *Sobic.004G164500* | 0.71 |
| *Sobic.003G260800* | 0.71 |
| *Sobic.003G136000* | 0.71 |
| *Sobic.003G115900* | 0.71 |
| *Sobic.003G033500* | 0.71 |
| *Sobic.002G407100* | 0.71 |
| *Sobic.002G368100* | 0.71 |
| *Sobic.002G301400* | 0.71 |
| *Sobic.002G271000* | 0.71 |
| *Sobic.002G232100* | 0.71 |
| *Sobic.002G203000* | 0.71 |
| *Sobic.002G187400* | 0.71 |
| *Sobic.002G185300* | 0.71 |
| *Sobic.002G182500* | 0.71 |
| *Sobic.002G181300* | 0.71 |
| *Sobic.002G145500* | 0.71 |
| *Sobic.002G115800* | 0.71 |
| *Sobic.002G079200* | 0.71 |
| *Sobic.002G024100* | 0.71 |
| *Sobic.002G014300* | 0.71 |
| *Sobic.001G450800* | 0.71 |
| *Sobic.001G436000* | 0.71 |
| *Sobic.001G160200* | 0.71 |
| *Sobic.001G146100* | 0.71 |
| *Sobic.001G022900* | 0.71 |
| *Sobic.001G021500* | 0.71 |
| *Sobic.001G003700* | 0.71 |
| *Sobic.010G107000* | 0.7 |
| *Sobic.010G094600* | 0.7 |
| *Sobic.009G213600* | 0.7 |
| *Sobic.008G187800* | 0.7 |
| *Sobic.008G103700* | 0.7 |
| *Sobic.007G212300* | 0.7 |
| *Sobic.007G147300* | 0.7 |
| *Sobic.006G171500* | 0.7 |
| *Sobic.005G077100* | 0.7 |
| *Sobic.004G083300* | 0.7 |
| *Sobic.004G041600* | 0.7 |
| *Sobic.004G039500* | 0.7 |
| *Sobic.003G374500* | 0.7 |
| *Sobic.003G243000* | 0.7 |
| *Sobic.003G049400* | 0.7 |
| *Sobic.003G049300* | 0.7 |
| *Sobic.002G346700* | 0.7 |
| *Sobic.002G117300* | 0.7 |
| *Sobic.002G009600* | 0.7 |
| *Sobic.001G492600* | 0.7 |
| *Sobic.001G469300* | 0.7 |
| *Sobic.001G083800* | 0.7 |

**Supplementary Table S5.** RNA-seq mapping rate.

| Series | Samples | Description | All reads | Maping reads | Mapping rate |
| --- | --- | --- | --- | --- | --- |
| GSE54705 | SRR1161660 | CK60-rep1 | 8043004 | 7426420 | 92.30% |
| GSE54705 | SRR1161661 | CK60-rep2 | 8090620 | 7223772 | 89.30% |
| GSE54705 | SRR1161662 | CK60-rep3 | 8181974 | 6601544 | 80.70% |
| GSE54705 | SRR1161663 | CK60-rep4 | 8935110 | 8117303 | 90.80% |
| GSE54705 | SRR1161664 | TX623-rep1 | 7676195 | 6941579 | 90.40% |
| GSE54705 | SRR1161665 | TX623-rep2 | 8153035 | 7622355 | 93.50% |
| GSE54705 | SRR1161666 | TX623-rep3 | 8448933 | 7424541 | 87.90% |
| GSE54705 | SRR1161667 | TX623-rep4 | 5413026 | 4646063 | 85.80% |
| GSE54705 | SRR1161668 | SCS-rep1 | 7898634 | 7048599 | 89.20% |
| GSE54705 | SRR1161669 | SCS-rep2 | 7020162 | 5933319 | 84.50% |
| GSE54705 | SRR1161670 | SCS-rep3 | 7065009 | 5483403 | 77.60% |
| GSE54705 | SRR1161671 | SCS-rep4 | 7867463 | 7014100 | 89.20% |
| GSE54705 | SRR1161672 | Ch17-rep1 | 7435200 | 6185889 | 83.20% |
| GSE54705 | SRR1161673 | Ch17-rep2 | 7144292 | 5600531 | 78.40% |
| GSE54705 | SRR1161674 | Ch17-rep3 | 7583410 | 6895437 | 90.90% |
| GSE54705 | SRR1161675 | Ch17-rep4 | 8620338 | 7937895 | 92.10% |
| GSE54705 | SRR1161676 | KS78-rep1 | 8585610 | 7912219 | 92.20% |
| GSE54705 | SRR1161677 | KS78-rep2 | 7980962 | 7212230 | 90.40% |
| GSE54705 | SRR1161678 | KS78-rep3 | 8262265 | 7511888 | 90.90% |
| GSE54705 | SRR1161679 | KS78-rep4 | 8246341 | 7562143 | 91.70% |
| GSE54705 | SRR1161680 | High Bulk-rep1 | 7794897 | 7088943 | 90.90% |
| GSE54705 | SRR1161681 | High Bulk-rep2 | 6962892 | 6020037 | 86.50% |
| GSE54705 | SRR1161682 | High Bulk-rep3 | 6233623 | 5097127 | 81.80% |
| GSE54705 | SRR1161683 | High Bulk-rep4 | 8326866 | 7472844 | 89.70% |
| GSE54705 | SRR1161684 | Low Bulk-rep1 | 8560496 | 7827367 | 91.40% |
| GSE54705 | SRR1161685 | Low Bulk-rep2 | 7770775 | 7048534 | 90.70% |
| GSE54705 | SRR1161686 | Low Bulk-rep3 | 8066953 | 7346477 | 91.10% |
| GSE54705 | SRR1161687 | Low Bulk-rep4 | 7229949 | 6588765 | 91.10% |
| SRP008469 | SRR349643 | 20 Day leaves | 24093871 | 25969963 | 95.80% |
| SRP008469 | SRR349644 | Emerging inflorescence | 27105721 | 23502539 | 96.40% |
| SRP008469 | SRR349645 | Seed 5 DAP | 24369809 | 29184607 | 95.50% |
| SRP008469 | SRR349646 | Early in florescence | 30553074 | 27931966 | 95.50% |
| SRP008469 | SRR349754 | Pistil | 29236733 | 27931966 | 95.50% |
| SRP008469 | SRR349767 | Embryo 25 DAP | 24728896 | 23677214 | 95.70% |
| SRP008469 | SRR349768 | Endosperm 25 DAP | 20541727 | 19238893 | 93.70% |
| SRP008469 | SRR349769 | Anther | 22880991 | 22007671 | 96.20% |
| SRP008469 | SRR349771 | seed 10 DAP | 26117975 | 25415570 | 97.30% |
| SRP008469 | SRR349772 | seed 20 DAP | 33131592 | 30772709 | 92.90% |
| GSE50464 | SRR959765 | Embryos_Sorghum | 13075113 | 11782938 | 90.10% |
| GSE50464 | SRR959766 | Floral_Merstem_Sorghum | 18323161 | 16743299 | 91.40% |
| GSE50464 | SRR959767 | Floral_Merstem_Sorghum | 17857474 | 15390881 | 86.20% |
| GSE50464 | SRR959768 | Flowers_Sorghum | 4191357 | 3382819 | 80.70% |
| GSE50464 | SRR959769 | Flowers_Sorghum | 17590207 | 14634585 | 83.20% |
| GSE50464 | SRR959770 | Flowers_Sorghum | 17703271 | 15592095 | 88.10% |
| GSE50464 | SRR959771 | Flowers_Sorghum | 16949257 | 15408623 | 90.90% |
| GSE50464 | SRR959772 | Vegetative_Meristem_Sorghum | 18523221 | 15874008 | 85.70% |
| GSE50464 | SRR959773 | Vegetative_Meristem_Sorghum | 18662433 | 16891895 | 90.50% |
| GSE50464 | SRR959774 | ABA_Sorghum_Root | 13676070 | 12657106 | 92.50% |
| GSE50464 | SRR959775 | ABA_Sorghum_Root | 8750120 | 7811897 | 89.30% |
| GSE50464 | SRR959776 | ABA_Sorghum_Root | 18153388 | 16701703 | 92.00% |
| GSE50464 | SRR959777 | ABA_Sorghum_Root | 19070668 | 17292418 | 90.70% |
| GSE50464 | SRR959778 | ABA_Sorghum_Root | 27954546 | 25480622 | 91.20% |
| GSE50464 | SRR959779 | PEG_Sorghum_Root | 18234434 | 17027880 | 93.40% |
| GSE50464 | SRR959780 | PEG_Sorghum_Root | 19390353 | 17987921 | 92.80% |
| GSE50464 | SRR959781 | PEG_Sorghum_Root | 28588473 | 27195574 | 95.10% |
| GSE50464 | SRR959782 | H2O_Sorghum_Root | 13234082 | 12312653 | 93.00% |
| GSE50464 | SRR959783 | H3O_Sorghum_Root | 10976946 | 9757730 | 88.90% |
| GSE50464 | SRR959784 | H4O_Sorghum_Root | 18298148 | 15447401 | 84.40% |
| GSE50464 | SRR959785 | H5O_Sorghum_Root | 19540019 | 18118999 | 92.70% |
| GSE50464 | SRR959786 | H6O_Sorghum_Root | 29856257 | 25856960 | 86.60% |
| GSE50464 | SRR959787 | NAOH_Sorghum_Root | 19182176 | 17532506 | 91.40% |
| GSE50464 | SRR959788 | NAOH_Sorghum_Root | 19947877 | 17125497 | 85.90% |
| GSE50464 | SRR959789 | NAOH_Sorghum_Root | 28935192 | 27314530 | 94.40% |
| GSE50464 | SRR959790 | ABA_Sorghum_Shoot | 17952846 | 16822272 | 93.70% |
| GSE50464 | SRR959791 | ABA_Sorghum_Shoot | 19047800 | 17898399 | 94.00% |
| GSE50464 | SRR959792 | ABA_Sorghum_Shoot | 27482043 | 25246199 | 91.90% |
| GSE50464 | SRR959793 | PEG_Sorghum_Shoot | 19722558 | 18805451 | 95.30% |
| GSE50464 | SRR959794 | PEG_Sorghum_Shoot | 19008253 | 17916581 | 94.30% |
| GSE50464 | SRR959795 | PEG_Sorghum_Shoot | 28517375 | 26337190 | 92.40% |
| GSE50464 | SRR959796 | H2O_Sorghum_Shoot | 19127234 | 16873661 | 88.20% |
| GSE50464 | SRR959797 | H3O_Sorghum_Shoot | 19275343 | 16319107 | 84.70% |
| GSE50464 | SRR959798 | H4O_Sorghum_Shoot | 27858903 | 23435774 | 84.10% |
| GSE50464 | SRR959799 | NAOH_Sorghum_Shoot | 16984925 | 15158857 | 89.20% |
| GSE50464 | SRR959800 | NAOH_Sorghum_Shoot | 24913612 | 22819112 | 91.60% |
| GSE50464 | SRR959801 | NAOH_Sorghum_Shoot | 28793016 | 25547696 | 88.70% |
